# Commercial provider staff experiences of the NHS low calorie diet programme pilot: a qualitative exploration of key barriers and facilitators

**DOI:** 10.1186/s12913-023-10501-y

**Published:** 2024-01-10

**Authors:** Susan Jones, Tamara J Brown, Patricia Watson, Catherine Homer, Charlotte Freeman, Chirag Bakhai, Louisa Ells

**Affiliations:** 1 School of Health & Life Sciences, Teesside University, Centuria Building, Middlesbrough, North Yorkshire TS1 3BX UK; 2 Obesity Institute, School of Health, Leeds Beckett University, City Campus, Leeds, LS6 3QW UK; 3 Sport and Physical Activity Research Centre, Sheffield Hallam University, Olympic Legacy Park, 2 Old Hall Road, Sheffield, S9 3TU UK; 4 Public Health Team, Calderdale Council, Princess Buildings, Princess Street, Halifax, West Yorkshire HX1 1TP UK; 5 Larkside Practice, Churchfield Medical Centre, 322 Crawley Green Road, Luton, Bedfordshire, LU2 9SB UK

**Keywords:** Diabetes, Obesity, Diet, Re:Mission, Normalisation process theory, Delivery of health care

## Abstract

**Background:**

The National Health Service Type 2 Diabetes Path to Remission programme in England (known as the NHS Low Calorie Diet programme when piloted) was established to support people living with excess weight and Type 2 Diabetes to lose weight and improve their glycaemic control. A mixed method evaluation was commissioned to provide an enhanced understanding of the long-term cost effectiveness of the pilot programme, its implementation, equity and transferability across broad and diverse populations. This study provided key insights on implementation and equity from the service providers’ perspective.

**Methods:**

Thirteen focus groups were conducted with commercial providers of the programme, during the initial pilot rollout. Participants were purposively sampled across all provider organisations and staff roles involved in implementing and delivering the programme. Normalisation Process Theory (NPT) was used to design the topic schedule, with the addition of topics on equity and person-centredness. Data were thematically analysed using NPT constructs with additional inductively created codes. Codes were summarised, and analytical themes generated.

**Results:**

The programme was found to fulfil the requirements for normalisation from the providers’ perspective. However, barriers were identified in engaging GP practices and receiving sufficient referrals, as well as supporting service users through challenges to remain compliant. There was variation in communication and training between provider sites. Areas for learning and improvement included adapting systems and processes and closing the gap where needs of service users are not fully met.

**Conclusions:**

The evaluation of the pilot programme demonstrated that it was workable when supported by effective primary care engagement, comprehensive training, and effective internal and external communication. However, limitations were identified in relation to programme specifications e.g. eligibility criteria, service specification and local commissioning decisions e.g. pattern of roll out, incentivisation of general practice. A person-centred approach to care is fundamental and should include cultural adaptation(s), and the assessment and signposting to additional support and services where required.

**Supplementary Information:**

The online version contains supplementary material available at 10.1186/s12913-023-10501-y.

## Introduction

In England, approximately 3.4 million people aged 16 years and over, live with Type 2 diabetes [1] and 64% of adults live with excess weight, 26% of whom live with obesity [2]. Urgent action is therefore needed to reduce the significant impact on health and wellbeing, and the associated NHS and societal costs [1, 3].

Recent evidence shows that a low-calorie diet (LCD) using Total Diet Replacement (TDR), can produce clinically significant weight loss, support remission of type 2 diabetes (T2D), and improve quality of life, for some people living with, or at risk of obesity and T2D [4–11]. The NHS Long Term Plan [12] made a commitment to pilot an LCD programme delivered using TDR, for people living with excess weight and T2D. During the initial 12-weeks of the programme, all meals are replaced with TDR products (e.g., soups and shakes) amounting to 800–900 kilocalories per day, followed by 6 weeks of food reintroduction and weight maintenance for a further 34 weeks, provided through behaviour change support.

NHS England (NHSE) initially piloted the programme across ten sites, each receiving one of three delivery models (group, 1:1 and digital) (see Additional File 1) [13]. The NHS LCD programme was delivered by commercial service providers, with the local health system (referred to hereon in as ‘primary care’) being responsible for referring eligible patients to the programme. Commissioning of commercial weight management programmes could be viewed as part of the process of ‘commercialisation of care’ within the NHS. The commercial providers are private limited companies which are for profit, and this context should be borne in mind when considering key insights on implementation and equity from the service providers’ perspective. The programme was available to adults (18–65 years) with a body mass index (BMI) ≥ 27 kg/m² (≥ 25 kg/m² for Black, Asian and other minoritized ethnic groups) and T2D diagnosis within the last 6 years [14]. The programme aimed to achieve remission from T2M by improving glycaemic parameters and weight loss. This programme has now been adapted following the evaluation process and renamed the NHS Type 2 Diabetes Path to Remission programme. The COVID-19 pandemic occurred concurrently with the planned implementation of the LCD programme pilot, so most of the delivery at the point of data collection had been remote.

The Re:Mission study [15] is the evaluation of the pilot NHS LCD Programme, and as part of this evaluation this paper aims to explore the experiences of provider staff to address the following research question:

*What do provider staff perceive to be the key barriers and facilitators to effective delivery, integration and normalisation into routine care?*

## Methods

This study received ethical approval from Leeds Beckett University (REF79441) and is reported using the COREQ checklist (see Additional File 2) [16]. Participants were purposively sampled across all four provider organisations and across all staff roles involved in implementing and delivering the programme across the first ten pilot sites in England. The link administrator in each organisation was contacted and they organised the focus groups. Researchers were not known to the participants. All eligible staff were invited to take part. Focus groups (n = 13) were conducted with staff (n = 66), two participants were unable to attend on the day, but one attended an alternative group. Two provider organisations took part in three focus groups each, one provider organisation took part in 5 focus groups and one provider organisation took part in 2 focus groups. Staff included senior and middle managers, and frontline staff in each organisation. Programme deliverers had a variety of experience and were either registered dietitians or nutritionists or had an undergraduate or postgraduate degree in nutrition, public health, sports exercise, or psychology. Some staff held multiple roles delivering and supporting delivery of the programme, and some programme deliverers also held management roles; many staff worked on multiple programmes within each organisation. Table 1 shows the characteristics of the focus groups.

**Table 1:** Characteristics of focus groups

Mode of programme delivery	Role of participants	Number of participants*
1 to 1	Programme deliverers	4
1 to 1	Programme deliverers	5
1 to 1	Senior & middle management team	5
1 to 1, Group, Digital	Senior & middle management team	6
1 to 1	Programme deliverers	5
1 to 1, Group, Digital	Patient support team	4
Digital	Digital team	6
Group	Programme deliverers	5
Digital	Senior & middle management team	8
Digital	Programme deliverers	8
Digital	Patient support team	6
Group	Programme deliverers	6
Group	Senior & middle management team	3

*n = 66 participants, 3 people participated in 2 focus groups each and 1 person participated in 3 focus groups (these 4 people had multiple roles within the organisation)

Normalisation Process Theory (NPT) was used to collect and analyse the data [17]. We used NPT to describe ‘a linear process in time’, starting with sense-making, then moving through engagement and participation, taking action and then reflecting back [18, 19]. NPT is flexible in how it can be used, which enabled us to conduct the analysis in response to what we were sensing in the data. Thematic analysis is a widely used method and was our starting point [20], using the main four NPT constructs (Coherence, Cognitive Participation, Collective Action, and Reflexive Monitoring) deductively, but also creating codes inductively, both within the NPT constructs and introducing additional codes. We broadly followed Thomas and Harden’s outline for thematic synthesis: using a 3-stage process of coding of text ‘line-by-line’; the development of ‘descriptive themes’; and the generation of ‘analytical themes’ [21]. These analytical themes can be equated to third order interpretations. Applying these systematic, in-depth, coding methods to the data increased the rigour and transparency of the analysis.

### Data collection

The focus group questions were designed to answer the research questions and were underpinned by NPT constructs (see Additional File 3). The question schedule was sent in advance and the first focus group acted as a pilot, whereupon minor amendments to the questions were made. Focus groups were conducted by researchers experienced in qualitative data collection (SJ, PW, CH, CF) between October 2021 and February 2022, and were one hour long. Most focus groups consisted of staff carrying out one role type; however, some were mixed. Focus groups were conducted online using MS Teams, audio and video recorded, and written observational field notes taken. Recordings were transcribed verbatim.

### Data analysis

#### Line-by-line coding

All transcripts were coded (TB, SJ, PW) using the framework described in Additional File 4. TB and SJ then coded the text in the main nodes to all the sub-nodes, some of which they created independently. NVivo software (QS International Pty Ltd. Version 12) was used to assist the process of storing and organising textual data and initial coding (See Additional File 4 for codebook).

#### Generation of descriptive themes

TB and SJ reviewed and streamlined the content of the nodes. TB and SJ reflected on the definitions of the node properties and refined the codebook, ensuring the sub-node labels and definitions accurately reflected the text contained within them. SJ reviewed the inductive nodes through the theoretical lens of NPT for interpretation and renamed some of them to reflect this change. Distinct inductive nodes and sub-nodes were kept, in addition to NPT components, and gaps in the data were identified. TB and SJ reviewed the spread of the data across the codebook both within and across provider type and staff role. TB and SJ completed narrative summaries for each node and wrote key summary statements, underpinned by quotations.

#### Generation of analytical themes

SJ used the summary statements to answer research question 1 by mapping out the data through the lens of the four NPT concepts of coherence, cognitive participation, collective action, and reflexive monitoring. TB and SJ inferred barriers and facilitators from what the participants had told us about their experiences (See Additional File 4). Through discussion, additional analytical themes were identified that contributed to the ‘strategic narrative’ [22] which underpinned the implications for intervention development and future implementation.

## Findings

The following section outlines the descriptive themes, followed by the analytical themes associated with barriers and facilitators that were inferred from the data coded to NPT constructs. Exemplar quotes are included; summary statements, data linkage tables, and quotations are in Additional File 5.

### Coherence

#### Barriers to coherence

Four main barriers to coherence were identified.

1) There was uncertainty between provider staff about whether the principal aim of the programme was diabetes remission or weight loss. Providers sometimes framed it from the service user (SU) point of view and recognised that the focus of the programme might differ between SUs. Providers cited other aims, including reduced blood glucose, blood pressure and medication. Long-term and holistic aims were to stabilise and sustain SUs improved condition and quality of life.

“I think sort of weight loss was kind of seen as a main sort of aim in general, so then use weight loss to then progress diabetes remission along the way, sort of stabilise you know, kind of help with people struggling with blood glucose levels. I guess education around sort of nutrition, lifestyle and building sort of a more sustainable healthy living aspect as well.” (PS1, PFG01).

2) The LCD programme was very similar in parts to other weight management programmes and sometimes used the same staff and systems.

“… I think the reality is when it comes to health care programmes the content isn’t massively different when the population is the same, so obviously there was that support that I could give as well with kind of the training and the learning aspect.” (PFG06, PS16).

3) The LCD programme was more onerous for staff compared to other weight management/ diabetes programmes. Providers noticed that the LCD programme was ‘our most complex programme’ (PS43) with ‘more serious data collection’ (PS32).

4) The LCD programme was reported as more challenging than other similar weight management programmes for SUs to remain compliant, in terms of intensity and duration.

“We’re obviously looking in greater depth about reducing meds, achieving remission, and so on so forth. So there is quite a big difference between what we’re trying to achieve. You know the programme’s longer, there’s more support, you know, intense is probably the wrong word, but the 12 weeks is quite intense for people. So that’s kind of the main differences between the two, I guess.” (PFG07, PS11).

#### Facilitators to coherence

Three main facilitators to coherence were identified.

1) The main difference to other programmes was the TDR phase which involves a complete removal of food for the first 12 weeks.

“…think the obvious massive difference is that you completely take solid food away from people, isn’t it? So it doesn’t really give them any, any choice but to focus on the reasons why. So that for me I think is, is personally the biggest difference.” (PFG02, PS5).

Providers also differentiated the LCD programme according to high level, longer-term support for SUs.

“And I think what I think I really like about this is not just the short-term fix which it does provide you help the diabetes go into remission, but also the longer term coaching support that they get provided with, which I think when I’ve worked in weight loss groups and exercise referrals and various things for many many years now, I think this is quite exceptional in terms of the way it delivers it.” (PS6, PFG02).

2) Providers being responsible for SU’s clinical measurements facilitates coherence by providing a satisfactory rationale for the role of the provider. SU self-monitoring of their clinical measurements also acted as a motivational tool for positive behaviour change.

“So basically we obviously have to collect the set data for every single session. So weight, glucose levels and then blood pressure if necessary.” (PG504, PS11).

“So taking that blood glucose and taking those readings from people is really effective.” (PFG07, PS30).

3) Effectiveness of the programme was confirmed to the providers by SU’s weight loss and reduced blood glucose and diabetes medications.

“… you get results much more quickly than in the NHS. I mean one of the biggest problems in the NHS, I was seeing patients twice a year, and you can’t get results that way.” (PFG11, PS50).

Following and supporting SUs in their personal journeys encouraged provider staff to buy-into the programme.

**“it’s quite rare as well for any sort of behaviour change programme, that you get to stay with someone for 12 months. So, it’s quite a unique opportunity, when you, when it’s a client led service, which is what it is.” (PFG01, PS4) **.

Four, inter-dependent, descriptive themes were identified: (1) preparation for mobilisation, (2) supporting enrolment and delivery, (3) communication and (4) training sessions. For details on themes 1 and 2 see Additional File 5.

#### Communication

Communication, feedback loops, and training, were crucial to engagement and participation with the programme, for all the stakeholders. Communication channels could be external or internal to the provider organisation. Middle managers played a vital role bridging communication between stakeholders and facilitating external training. Teamwork and peer support was important in facilitating cognitive participation.

#### Feedback loops

Feedback was used by provider organisations for service improvement, maintaining standards, encouragement of staff and professional development.

This coach recognised how feedback on delivery performance helped them to deliver a better service.

“So it goes both ways, it’s the support to SUs, the business to be able to make sure the delivery is correct and on how it’s done, and then any feedback on that, and to develop that, and then of course your own personal development in making sure that you’re doing [it right], you know, this is why we’re here enjoying our role to be able to support the SUs going forward.” (PFG04, PS19).

Routine data collection was also used to offer occasional feedback to the staff on progress of the pilot programme. This was eagerly awaited, “Cause obviously we’re waiting for that initial data to come back.” (PFG02, PS8).

Coaches also found feedback on the programme useful to answer SUs’ questions and to close feedback loops with other SU queries.

“… participants [SUs] ask a lot like how can you tell me how it’s going? […] being able to go back to him and say, look, average client loses nine and half kg?” (PFG02, PS5).

Communication loops were said to be present for negative/positive feedback and health incidents. When feedback was received from other stakeholders there was a process for it to be disseminated appropriately. If it was positive, it was shared with the team lead and cascaded to team members.

“… we’ll share positive feedback, we will contact the delivery coach’s line manager to share positive feedback, to make sure that then gets shared with them. ‘Cause it’s important that, as line managers, they are aware of their team’s success and feedback that’s been provided.” (PFG05, PS21).

If feedback was negative, it was shared with the team lead and shared with team member(s) as appropriate, and followed up, where necessary.

“… in the event we were to get in negative feedback, again we’d follow a similar process so, depending on what the feedback was as well, we would speak to the coach’s line manager or if it was a patient support team, speak to their line manager, or it was to get escalated to myself [senior manager] to investigate, and this could be something like just looking at a training need or it could be absolutely anything.” (PFG05, PS21).

#### Training sessions

Provider organisations developed a schedule of training sessions for different audiences e.g. referrers, programme deliverers. There were induction and ongoing training sessions to ensure staff were able to fulfil their functions and specific training where a skills need was identified (see Additional File 5).

#### Facilitators and barriers to cognitive participation

Effectiveness of these communication channels and training sessions was important, as they had the potential to become facilitators or barriers to engagement and participation.

#### Facilitators to communication

Open channels were the basis for communication and require fully operational, complete feedback loops, with efficient and effective communication links that function well.

“… we’ll just ask a question on there (coaches’ WhatsApp), or just email another coach and say, do you know anything about this? Have you got any resources?” (PFG2, PS8).

Providers understood the link between acting on feedback and SU benefit and communicating externally with referrers and commissioners.

#### Facilitators to training

Provider staff recognised the importance of a standard schedule of teaching sessions, using multiple teaching methods, provided by experts within the provider organisations. Training was ongoing and responsive to the changing needs of staff, who gave enthusiastic and positive feedback about the training programme.

“I think all those [elements of the programme] are covered to a really good depth in terms of the, the training cycle and that means that when you do go out into the field you feel well prepared and organised.” (PFG02, PS06).

Participants reported that when quantitative data were triangulated with personal observation, it was compelling and reinforced engagement because it demonstrated that what they learnt in training appeared to be effective.

“I think it’s really positive the feedback that participants are giving to coaches and, and what coaches are seeing in terms of changes with participants and also from a data aspect that we tend to pull, you can see that it’s being beneficial to people.” (PFG13, PS63).

#### Barriers to communication

When communication channels became blocked this created a barrier. This could happen when feedback loops were broken or incomplete, or the links in other communication channels were dysfunctional. Provider staff felt this posed a barrier to offering high quality care. Coaches noticed how communicating across organisational boundaries was harder than within the provider organisation, and that having more responsibility held within the provider organisation, eased some of these challenges.

“I mean, I don’t know about other organisations, but I think when I speak from like primary care setting there is sometimes a bit of a disconnect. That’s not in a, it’s just sometimes the two don’t always marry up. I feel that LCD is a bit better because if the responsibility is given to us more, which I think is better, but I feel like when the, when there’s a bit more responsibility on the primary care network, it sometimes it’s a bit disjointed… [holding more responsibility within the provider] does relieve a lot of pressures.” (PFG06, PS26).

#### Barriers to training sessions

There were examples of increased workload, as new training sessions had to be developed and standardised, and with the creation of additional material required as the programme became more established. GP involvement in training differed across pilot areas (see Additional File 5, Mobilisation) and providers tried to adapt the training material accordingly, however, this created an additional burden on resources. Healthcare staff had to be persuaded on the benefits for specific patients, and the difference between the LCD and other programmes. Providers referred to the need to train GPs in the accurate identification and referral of suitable patients.

“So we go through that in a lot of detail explaining the referral form process with them and the pathway.” (PFG10, PS49).

### Collective action

#### Barriers faced by service users

The main barriers for SUs appeared to disproportionately impact people from minoritised ethnic groups (people from Asian or African Caribbean backgrounds were mentioned by provider staff). Multiple intersecting barriers related to language and the need for translation, but the minoritised ethnic group was not specified.

“I would say from my perspective, one of the challenges is the fact that we can’t offer the care in, in any language other than English unless the patient provides their own translator. So if someone hasn’t got a family member or friend who’s willing to translate for them for the duration of the programme, then we can’t accept them onto the programme, so obviously that has its issues. And I think for a 12-month programme it’s quite a lot for a patient to depend on a family member or friend to be able to commit to that translation for a long period of time, so that’s definitely a barrier.” (PFG12, PS60).

Lack of family support was reported as a barrier which more commonly affected women from minoritised ethnic groups, who reported challenges in managing the programme whilst still cooking for their families.

“Yeah no I was just thinking about my experience and I, I think I had very few patients coming from Asian or African Caribbean background, very few patients. And I noticed that with some of these patients, especially if they are women there is like, like a big challenge, is that some time they are not really understood or supported from their family because traditional food is such a big part of their culture, especially women, they are the main cooker in the house. So one of the main challenges to discuss is how they’re going to still, you know, cook and provide for their families, for their husband. So sometimes they are not as much understood or supported from, from their, from their families. But not all the time, but it’s something that I found more common from, from this patient coming from these backgrounds, I think.” (PFG11, PS51).

Lack of attention to traditional cultural events such as Ramadan and Christmas, especially during the TDR phase, can create a barrier.

If TDR was not free of charge, provider staff speculated that this would be a key barrier.

“We do get people asking though if, is it, is it free, free of charge before we’ve had time to tell them it’s free of charge, so they do always ask that. A few do always ask that question, so it could be a barrier.” (PFG05, PS23).

Provider staff viewed managing conversations about mental health as a barrier to covering intended programme content; these conversations were not given enough coverage within the sessions.

“…and obviously as well like I said before, a lot of emphasis on the mental health side of things. You know I might have had a topic planned or something planned for that, for that session, but then conversation’s taken a bit of a turn so we’ve, you know, delved a bit deeper into that side of things. So yeah, I think, I think especially I don’t know about anyone, anyone else, but I’d say especially with my participants there’s, there seems to be a lot of mental health conversations going on there.” (PFG02, PS9).

Lack of trust could be a barrier to engagement, regardless of method of programme delivery.

“So we, we end up, we work with, it’s the same as someone in a traditional environment coming to your clinic with their arms crossed and they sit there and they’re yes, no answers. We use the same skills, just in a, in a, in a remote setting really.” (PFG11, PS57).

There were many personal characteristics and circumstances of SUs that providers felt created barriers to completing the programme. Reasons were inconsistent and highlighted the multiple and varied reasons for drop-out, which are unique to each SU. Reasons included psychological reasons, chaotic circumstances, multiple life events, busy lifestyles, work commitments that revolve around food, people with larger families, people who do not work but have a lot of ‘thinking time’, living with severe depression and other health issues, having a lot going on at home, mental traumas, and mindset.

“For me, my group has been the workers tend to be more disciplined I found so they’re busy so they’re not likely to think, think about food as much. And I found that they get a lot of family support. And it tends to be people that don’t work who are left, who have got a lot of thinking time and got, got quite a lot going on at home or are quite severely depressed and got, got other issues and probably other health issues as well. They, they really want to do it at the beginning and I find it’s that type of person where it’s more likely to, to slip.” (PFG02, PS8).

Providers highlighted the importance of motivation to engage with the programme.

“Retention is better when GPs only refer those most likely to stay in the programme. So if the GPs do a good job of filtering and just kind of assessing who they think is suitable, explaining implicitly what it involves, then retention is going to be better and suitability for the programme is going to be higher.” (PFG04, PS17).

Providers highlighted cases they considered were ‘inappropriate’ referrals, suggesting a tension between referrers and providers over responsibility for retention.

“I’ve had one client who probably, who shouldn’t have been referred by their GP because of their mental health status in relation to food. So, there’s been previous disordered eating that wasn’t really, probably picked up and wasn’t appropriate for the referral in the first place.” (PFG01, PS4).

#### Facilitating referrals

Providers were ultimately reliant on referrers for workability of the programme. Low volume referrals made it challenging for providers to fill the spaces. It was suggested that more written information on the eligibility criteria and the risks from undertaking TDR, would help with the flow of referrals.

Providers viewed their role as bridging between stakeholders, to support the referral process. For example, contract liaison officers supported the liaison between SUs, their own organisations and the local health service leads. Therefore, good communication, including feedback and sharing of information, between contract liaison officers and GP practices, was deemed vital in making the programme work.

“Yeah I’d say so yeah, there’s the contract liaison officers there, the guys in the middle that will kind of liaise with the GP practices and then, and share information as they need to, same as we would, if we felt there was a GP practice that was incorrectly referring, as an example, we would feed that back to the CLO for that area, who would then work with that GP practice to make sure going forwards that, you know, the referrals that they sent through are eligible or correct.” (PFG05, PS21).

This bridging role reduced barriers and increased facilitators to programme activity. Achieving referrals required engagement with various primary care stakeholders (pharmacists, practice nurses).

“…I regularly meet with different practices, engage with different health groups of health professionals and this is often with in combination with our project officer within the CCG. So, we’ve been having a lot of different interactions. Some of them have been requested by referring pharmacists early on in, in the referral phase and a lot of the time it’s proactive with us, and it’s come sometimes through individuals wanting to be referred by GPs as well. So, there’s been an approach where we have addressed different practices and had significant volumes through from them, volumes of referrals, once we’ve taken that approach.” (PFG09, PS39).

However, providers relied mainly on communication with non-clinical staff in GP practices.

“I’d say mainly like the secretaries and the admin team at the, at the GP surgeries is who we speak to. Occasionally if we’ve been chasing something up and we’ve rang a practice a couple of times, we sent a few emails, we might get a nurse or GP calling if they’ve got a specific query, but generally it is just kind of their admin and secretary team that we speak to.” (PFG12, PS60).

### Reflexive monitoring

#### Requirements for normalisation

There emerged two strands to understanding the requirements for normalisation: those within the delivery organisation and those within the wider context. The embedding process within the provider organisation was evidenced through their communication structures.

“… pretty much weekly meetings, to sort of get to a point where, you know, we were all clear on what the plan was going to be and then reviewing those plans ongoing”. (PFG03, PS15)

There was recognition of the ongoing process of embedding knowledge and understanding of the programme within General Practice.

“I think it’s about general practice gaining familiarity, really understanding the programme. The GPs that have seen the individuals that have benefited, then go on to make the best next referrals”. (PFG09, PS39)

There was a need for key partners to build up confidence in the programme.

“I think one of the challenges is just per area primary care, understanding the level of all the different options and how this programme would then roll out is how does it sit alongside all the other, you know, programmes that an individual could qualify for multiple in the same time, and it’s supporting primary care to understand which one is going to be the most beneficial for each patient.” (PFG01, PS13).

There was reference to the cost effectiveness focus for the commissioners.

“… depends on the commissioning model, certainly you would need to get, you know, slightly higher volumes to make it particularly attractive and worthwhile to commission, let alone deliver”. (PFG09, PS41)

Links and contracts with other programmes were seen to strengthen the position of the provider organisation with the wider stakeholders.

“we’ve been commissioned separately by a local authority to deliver some weight management, Tier 2 weight management programmes, so we’re getting sort of the, as an organisation we’re getting slightly more embedded into delivery of related services, but those both complement each other”. (PFG09, PS41)

The requirements for normalisation were identified as good communication systems, that included informal and formal sessions with coaches and management, laying the foundation for increasing referrals and the belief that if the programme was successful it would mean further funding to extend the programme.

### Areas for improvement

#### TDR products

Providers varied in how many TDR products they offered. Provider staff suggested offering a wider choice of products and enabling SUs to taste before ordering. Provider staff said there was little scope for changing products if SUs did not like them and identified that the process for exchanging products needed improving.

“… they would really like to try products beforehand.” (PFG01, PS1).

“… about 20% that maybe of my participants have found that they really don’t like a certain flavour, or they’ve really struggled with that, and then it’s been kind of the, oh you’ve got to push through or order early and kind of hope they’ve got leftovers”. (PFG01, PS1)

#### Internal feedback

Provider staff identified that regular (at least monthly) feedback and updates needed to be prioritised, although the difficulties of bringing everyone together for feedback and update sessions was acknowledged, and one-to-one sessions suggested for those unable to attend. One coach said:

“A little bit of feedback [from management] would be a bit more appreciated but recognise time is an issue.” (PFG01, PS01).

#### Wider system issues

Wider system issues for providers ranged from national commissioning decisions related to the service specification, to local commissioning related to the area, through to receiving appropriate referrals in sufficient numbers and external factors, such as media portrayals of the programme, that influenced uptake. Nationally, there were requirements for eligibility, such as having had retinal screening, that led to exclusion. Staff reported that locally, to be able to make well informed decisions, commissioners needed to see and understand how the programme fitted with what else was going on in the area.

**“**I think from a commissioning point of view it’s, it’ll probably be seeing how it fits as part of a wider sort of offer for the, for the, whichever area the program runs in, so you have different levels of service, like NDPP, and you have the, obviously the low calorie diet programme, but then you’ll have so also other Tier 2 services, and commissioners will be thinking about that balance between what’s on offer between those different services.” (PFG01, PS04).

In the opinion of provider staff, the referral process required improvement e.g. simplifying, offering financial incentives to GPs for referrals. Increased confidence in, and understanding of the programme, were expected to improve referral rates. Inappropriate referrals, particularly early on, showed a lack of clarity on what the programme could offer and who would benefit most. Staff noted how it took time to build up trust with GPs and for them to build confidence in the programme– especially when requesting a change in medication.

“Convincing some practices about the meds changes required...These people [clients] have been on blood pressure meds for 10 years and suddenly we’re proposing just stopping them for example, and that takes a little bit of trust building.” (PFG12, PS57).

The boundaries around medical responsibilities were reported as contested e.g. when the provider requested the doctor to reduce the patient’s medication. Having a Medical Director (with appropriate clinical qualifications) within the provider organisation, to discuss issues around medical conditions and emerging issues, such as raised blood pressure and glucose readings, was seen as beneficial. From a provider perspective, relieving the GP practices of some of their responsibilities, should be seen positively as a resource saving.

Provider staff recognised that eligibility criteria were perceived as unnecessarily limiting by GPs. They suggested that eligibility processes needed to be revised to help increase numbers of referrals. Databases holding pilot data aided analysis of progress and outcomes; however, it was noted by one provider that their database would benefit from being able to store more qualitative information, such as patient lifestyle, family life, likes and dislikes.

#### Equity across SU population

One provider talked about how they were using their data to check for equity of referral; although this might have been happening it was not mentioned by other providers when the topic of equity was raised.

“… we would benchmark the referrals and the number of people that we had on the programme against the kind of local data. So, looking at IMD deciles, people you know ethnicities and gender, that kind of thing. And just to make sure that we were kind of, our referrals were representative of the local area”. (PFG10, PS49)

There was limited evidence that all providers were targeting the programme to ensure that it was being offered and accessible to all patients who met the criteria, taking into account SUs’ culture, religion or beliefs.

“… some of the external triggers, the examples that we had were like you know going to the pub or going out for a drink and that sort of thing that wasn’t quite appropriate for my [Asian ladies] group. So, I changed it slightly”. (PFG08, PS34)

Efforts to ensure the inclusion of minoritised ethnic groups was limited, with only one provider planning to make specific adaptations for those where English was not their first language.

“… maybe either visuals or, better visuals or in their language, you know, if it’s a common language that we can put it in that”. (PFG03, PS30)

“… we do have kind of Urdu speaking and Hindi speaking sessions coming up. So for those specific groups that we’re putting on in Feb, there will be a translated book hopefully for them”. (PFG03, PS11)

There appeared to be little recognition that more proactive work needed to be done to improve equity, rather than framing equity as a capacity issue or a lack of need.

“… it’s about practices’ capacity to do some of that targeted work so you know, if we know that, you know, a certain demographic is going to benefit more from it then doing that targeted work is sometimes what they’re not able to have that sort of time and capacity to do that “. (PFG03, PS15)

“… we have to you know, promote it and target those particular groups as I say and we, we’re working on a language group in November, but I don’t think we would have the demand unless we go out and promote it, specifically.” (PFG03, PS15).

Provider staff pointed out that more flexibility in delivery methods offered greater possibility of equity. Specifically, they noted how virtual delivery was preferred in many cases.

“20% virtual would be much more, much better from all the points you’re talking about around equity and inclusion and personalisation and all of that, let alone mobilisation and delivery”. (PFG09, PS41)

## Discussion

We have provided insights from commercial providers’ experiences of delivering the NHS LCD pilot programme. A strength of the study was that our sample was comprehensive and representative of staff roles, geographical regions, and delivery models across the four provider organisations, with only one participant not attending a focus group. The overall strategic narrative was one of ‘commercialisation of care’, meaning the structures and assumptions of commissioning, referral and provision operated through commercial contracts that do not reward spending extra resources on creating an equitable referral and delivery process. Provider and primary care structures and systems that facilitated coherence and promoted communication and training were more likely to lead to successful implementation, whilst acknowledging the importance of SU characteristics.

There is limited evidence of health care professionals’ perceptions about referring people for obesity care in community settings, or commercial providers perceptions of delivery of weight management, it is the latter which this paper seeks to redress [23]. Excellent communication between referrers and providers was fundamental; to engage GP staff and to train them in the referral process and follow-up communications of their patients. However, open communication channels with GP services were not always evident. This posed challenges for providers due to their dependence on receiving referrals from GP practices and also due to the responsibility for practices to provide ongoing support to SUs. This highlights commonality with the data from the local health service leads, which showed that engagement was dependent on the primary care team and collaboration was crucial to success of the programme [24].

Facilitators of *collective action* identified by providers included close teamwork within provider organisations, trusting relationships between coaches and SUs and wider choices of TDR products. This is in line with other literature around the importance of trust in the relationship between provider and SU [25]. A range of barriers were identified by providers for SUs compliance and continuation with the programme, associated with personal characteristics, environment, skills and preferences. The main barriers for SUs appeared to disproportionately impact people from minoritised ethnic groups. Multiple and intersecting barriers included ethnicity, culture, language and translation requirements, digital competency, veganism, taste preferences, needs of the family, and time to attend sessions. Language barriers puts the onus on and creates inequalities for the participant. Research by Digital Scotland highlights that language and translation requirements is the biggest barrier for ethnic minorities accessing weight management services [26]. The potential cost of TDR products outside of a pilot context was raised as a crucial barrier to collective action.

*Reflexive monitoring* highlighted areas of learning related to the practical implementation of the programme, motivation of SUs, and the importance of normalisation. Overall, provider staff reflected very positively about the benefits of the LCD programme and the ways it had been facilitated. From their perspective, what had been learnt was being used to adapt provision. Areas for improvement included internal feedback, wider system issues relating to referrals, TDR products and equity. Providers were concerned with getting the ‘right’ people referred into the programme, meaning motivated people and people without psychological issues related to food. There is a danger that this approach could increase inequity as the programme expands. It will be interesting to triangulate these findings with data from SUs (currently being collected).

Cross-cutting themes emerged from the data, namely equity and person-centredness. Equity of opportunity was missing, which is fundamental to address the social gradient in health [27]. It is highly likely there are unaddressed needs of individual participants who identify with minoritised groups,, that need to be met in order for these individuals to be able to achieve the same outcomes. Equity themes related to culture, ethnicity, language, and digital engagement. There was little evidence about inequities related to disability, age and place and no mention of sexual orientation or gender identity. Providers viewed non-English language as the main barrier to the programme being equitable. Equity is discussed in more depth in the local health service lead data [24].

Reflecting on the pilot, it was clear to providers that there was a limited response to adapting delivery for SUs from minoritized ethnic groups. Providers varied in their approach to cultural adaptations to the programme. A recent review of qualitative studies around uptake and adherence to weight management and T2D programmes highlights limited UK data with participants’ discourse around surface level factors such as language [28]. Barriers to engagement and adherence for minoritised ethnic groups is a significant barrier to equity. A recent documentary review of service parameters and behaviour change content across four independent service providers of low-calorie diet programmes, conducted as part of this Re:Mission study, highlights that healthy eating and cultural tailoring are essential components of the service specification and lack of cultural adaptation could have consequences for the success of minority group members of the programme [29].

Underlying psychological issues such as mental health and emotional eating were raised by providers and have been highlighted as an area of concern by tier 2 weight management SUs [30]. Emotional factors that impact on care must be considered if we are to deliver person-centred care [31], as SUs value weight management services that recognise the emotional aspect of weight management [32]. More work is required to understand these needs and the support (experience/skills/training) required to appropriately address them, either within the service or through signposted services.

Person-centred themes related to the importance of the coach and SU relationship, and providers being able to tailor the programme for the individual SU; the benefit of peer support between SUs was also recognised. This thread ran through the analysis, with buy-in from providers coming from building a relationship over time, being able to follow SUs throughout their journey, and observing the physical and psychosocial benefits, which reinforced providers’ coherence and positive perceptions of the programme. This thread stresses the importance of person-centred care and highlights some of the challenges of delivering this within the context of commercialisation of care.

### Limitations

We did not distinguish between the different providers when reporting the findings. As provider leads acted as the gatekeeper to staff, this may have impacted participation. There was also variation in staff roles within and between provider organisations, and some focus groups consisted of staff who line managed others in the group. This may have impacted on the openness of the discussion, as researchers noted that a positive perspective was given to all comments. It was noteworthy that no significant negatives were acknowledged, even when prompted by the researcher. It is therefore unclear as to whether responses were biased or reflected genuinely high levels of buy-in and operationalisation.

### Recommendations

Key learning points that facilitate remote delivery of the programme include:

- Taking a person-centred approach is critical and can be achieved by developing positive working relationship between provider and SU, which includes building trust, taking time, positive encouragement, constructive feedback, developing confidence, and signposting to additional support where required.
- There needs to be a thorough assessment of SU commitment to and interest in the programme by a clinician who is well informed about the specific offer; including SU eligibility, tolerance to the products, and medication changes at point of referral. More background information about each SU would help the delivery staff develop person-centeredness.
- Cultural adaptation of the programme should be continuously reviewed to ensure services meet the needs of target populations. Equality impact assessments would help providers to monitor equity in uptake and impact.
- Delivery team training in recognising and signposting support for mental health and emotional/disordered eating is important together with appropriate training.
- Provision of a diverse range of delivery methods is key to making the programme accessible and sustainable. One-to-one programme delivery enables providers to start delivery promptly, whereas group delivery is reliant on sufficient referral numbers which can delay the start date. Remote delivery enables providers to support more SUs. Supporting Apps can enable regular and timely feedback and support between provider and SUs. Providing a range of TDR products is important to SUs.
- An effective operational system is critical given the complexity of the programme and required data capture. Close and supportive teamwork is highly valued: access to immediate frontline support from colleagues using online chats during live coaching sessions with SUs was considered helpful.
- It is important to understand local care pathways and the wider context of the service delivery. High engagement by primary care and effective communication between providers and referrers is critical.

## Conclusions

The LCD programme pilot was most workable when supported by effective primary care engagement, comprehensive training of referrers and deliverers, and effective internal and external communication. However, provider organisations faced challenges working within the local context, where commissioners were applying a national, standard contract and service specification. A person-centred approach to care is fundamental and should include cultural adaptation and assessment and signposting to additional support and services where required.

### Electronic supplementary material

Below is the link to the electronic supplementary material.

Supplementary Material 1: Additional File 1 presents an overview of pilot areas, delivery models and programme structure

Supplementary Material 2: Additional File 2 provides a completed COREQ checklist

Supplementary Material 3: Additional File 3 provides information on NPT and the focus group question framework

Supplementary Material 4: Additional File 4 provides the codebook and an example of data linkage

Supplementary Material 5: Additional File 5 provides further findings and quotations from the data

## Data Availability

The datasets generated during this current study are not publicly available due to reasons of privacy and confidentiality, and because of the inability to de-identify the data. Additional knowledge of the data can be available from the corresponding author on reasonable request.

## Declarations

The views expressed in this paper are those of the author(s) and not necessarily those of the NHS or the National Institute for Health Research.

## Abbreviations

BMI: Body mass index
GP: General practitioner
LCD: Low Calorie Diet
NHS: National Health Service
NHSE: NHS England
NPT: Normalisation Process Theory
PFG: Provider focus group
PS: Participant
SU: Service user
TDR: Total Diet Replacement
T2D: Type 2 Diabetes
