# Supplementary material for: Commercial provider staff experiences of the NHS low calorie diet programme pilot: a qualitative exploration of key barriers and facilitators
Source: BMC Health Serv Res. 2024 Jan 10;24:53. doi: 10.1186/s12913-023-10501-y (PMC10782528; doi:10.1186/s12913-023-10501-y)
Supplement: Supplementary file 1 — Supplementary Material 1: Additional File 1 presents an overview of pilot areas, delivery models and programme structure [file 12913_2023_10501_MOESM1_ESM.docx]

**Additional File 1**

# Overview of the first 10 localities commissioned by NHS E

| **Localities** | **Delivery Model** |
| --- | --- |
| Bedfordshire, Luton and Milton Keynes | Digital |
| Birmingham and Solihull | Group |
| Derbyshire | Group |
| Frimley | 1:1 |
| Gloucestershire | Digital |
| Greater Manchester | Group |
| Humber Coast and Vale | Digital |
| North Central London | Digital |
| North East London | Group |
| South Yorkshire, and Bassetlaw | 1:1 |


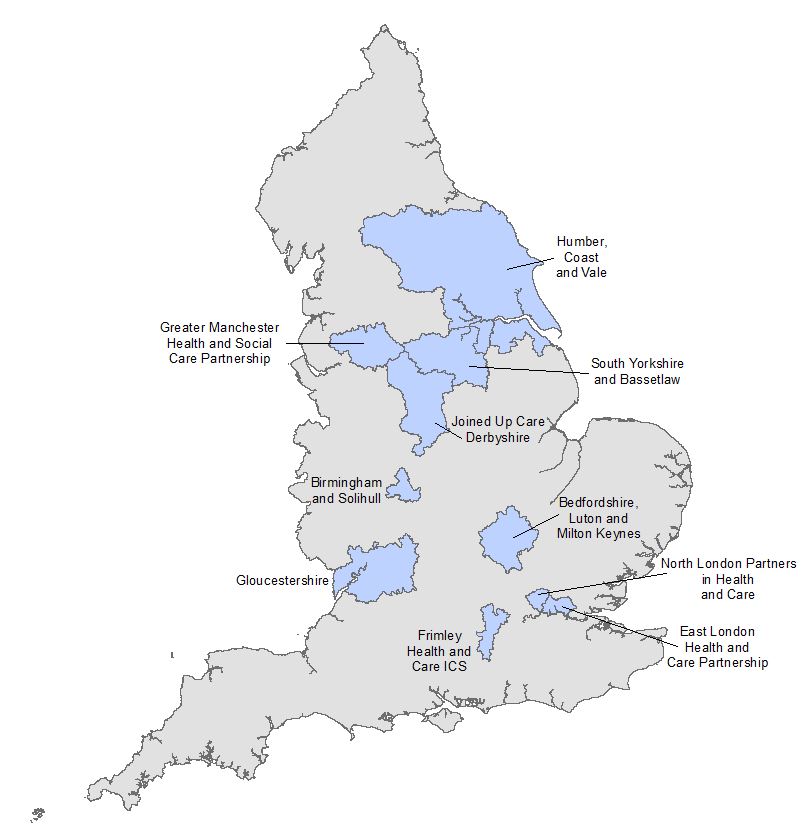


Geographical locations of the ten pilot sites; updated to April 2021 Integrated Care System configurations: taken from the Strategic Health Asset Planning Evaluation tool (SHAPE)

# The NHS Low Calorie Diet Programme Delivery Structure


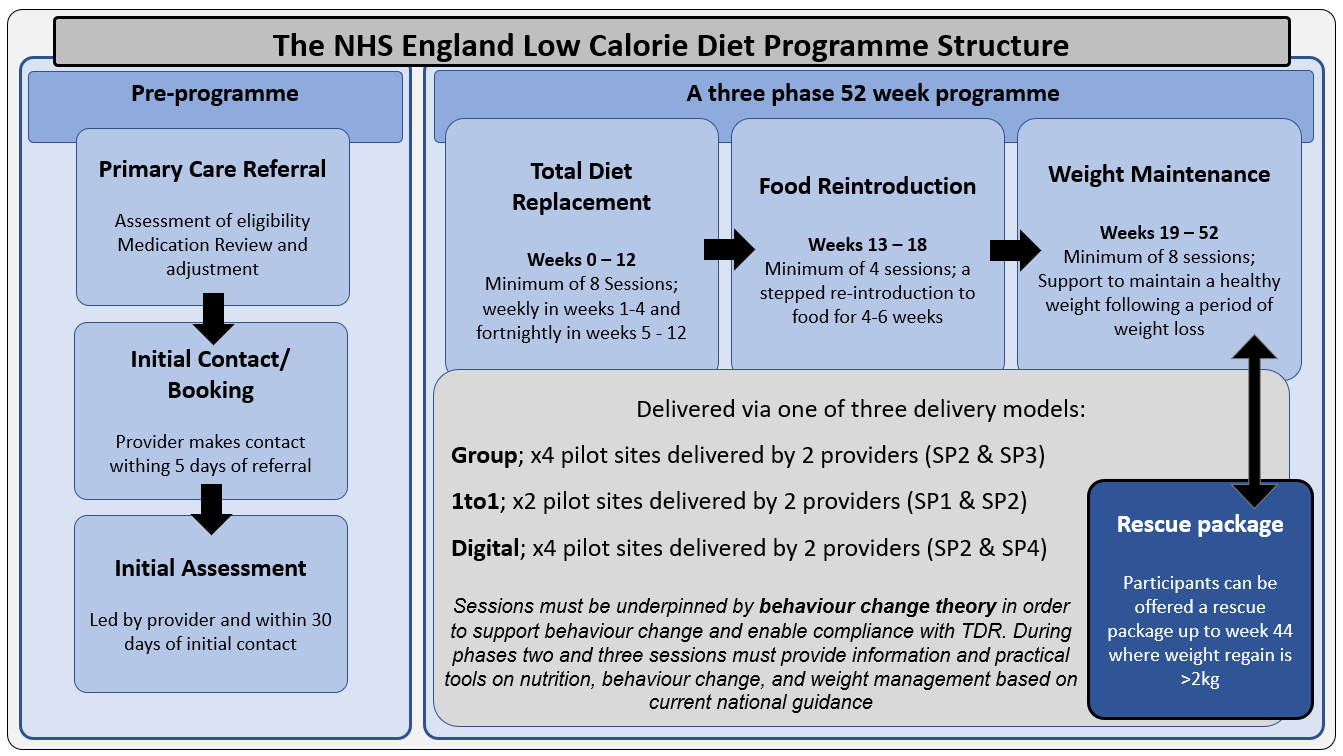


The four providers used different TDR product brands with large difference in range of products and flavours available. One provider provided 6 different options (soups and shakes) while a second provided 89 different options (soups, shakes, smoothies, bars, breakfasts, and pre-prepared meals). The other two providers provided 15 (soups, shakes, smoothies, and porridge) and 7 (soups, shakes and bars) options respectively.
