# Supplementary material for: Commercial provider staff experiences of the NHS low calorie diet programme pilot: a qualitative exploration of key barriers and facilitators
Source: BMC Health Serv Res. 2024 Jan 10;24:53. doi: 10.1186/s12913-023-10501-y (PMC10782528; doi:10.1186/s12913-023-10501-y)
Supplement: Supplementary file 2 — Supplementary Material 2: Additional File 2 provides a completed COREQ checklist [file 12913_2023_10501_MOESM2_ESM.doc]

**Additional File 2**

**Consolidated criteria for reporting qualitative studies (COREQ): 32-item checklist**

| **No. Item** | **Guide questions/description** | **Reported on Page #** |
| --- | --- | --- |
| **Domain 1: Research team and reﬂexivity** |  |  |
| *Personal Characteristics* |  |  |
| 1. Interviewer/facilitator | Which author/s conducted the interview or focus group? | pp.6 |
| 2. Credentials | What were the researcher’s credentials? E.g. PhD, MD | Additional File 2 |
| 3. Occupation | What was their occupation at the time of the study? | Additional File 2 |
| 4. Gender | Was the researcher male or female? | Additional File 2 |
| 5. Experience and training | What experience or training did the researcher have? | Additional File 2 |
| *Relationship with participants* |  |  |
| 6. Relationship established | Was a relationship established prior to study commencement? | pp.31 (acknowledgements) |
| 7. Participant knowledge of the interviewer | What did the participants know about the researcher? e.g. personal goals, reasons for doing the research | pp.4 |
| 8. Interviewer characteristics | What characteristics were reported about the interviewer/facilitator? e.g. Bias, assumptions, reasons and interests in the research topic | pp.6 |
| **Domain 2: study design** |  |  |
| *Theoretical framework* |  |  |
| 9. Methodological orientation and Theory | What methodological orientation was stated to underpin the study? e.g. grounded theory, discourse analysis, ethnography, phenomenology, content analysis | pp.6 |
| *Participant selection* |  |  |
| 10. Sampling | How were participants selected? e.g. purposive, convenience, consecutive, snowball | pp.4 |
| 11. Method of approach | How were participants approached? e.g. face-to-face, telephone, mail, email | pp.4 |
| 12. Sample size | How many participants were in the study? | pp.4, Table 1 |
| 13. Non-participation | How many people refused to participate or dropped out? Reasons? | pp.4, 24 |
| *Setting* |  |  |
| 14. Setting of data collection | Where was the data collected? e.g. home, clinic, workplace | pp.6 |
| 15. Presence of non-participants | Was anyone else present besides the participants and researchers? | N/A |
| 16. Description of sample | What are the important characteristics of the sample? e.g. demographic data, date | pp.5,6, Table 1, Additional File 1 |
| *Data collection* |  |  |
| 17. Interview guide | Were questions, prompts, guides provided by the authors? Was it pilot tested? | pp.6 and also  Additional file 3 |
| 18. Repeat interviews | Were repeat inter views carried out? If yes, how many? | N/A |
| 19. Audio/visual recording | Did the research use audio or visual recording to collect the data? | pp.6 |
| 20. Field notes | Were ﬁeld notes made during and/or after the interview or focus group? | pp.6 |
| 21. Duration | What was the duration of the interviews or focus group? | pp.6 |
| 22. Data saturation | Was data saturation discussed? | N/A |
| 23. Transcripts returned | Were transcripts returned to participants for comment and/or correction? | N/A |
| **Domain 3: analysis and ﬁndings** |  |  |
| *Data analysis* |  |  |
| 24. Number of data coders | How many data coders coded the data? | 2 (SJ and TB) |
| 25. Description of the coding tree | Did authors provide a description of the coding tree? | Additional File 4 |
| 26. Derivation of themes | Were themes identiﬁed in advance or derived from the data? | pp.6,7 |
| 27. Software | What software, if applicable, was used to manage the data? | pp.7 |
| 28. Participant checking | Did participants provide feedback on the ﬁndings? | N/A |
| *Reporting* |  |  |
| 29. Quotations presented | Were participant quotations presented to illustrate the themes/ﬁndings? Was each quotation identiﬁed? e.g. participant number | Yes, throughout |
| 30. Data and ﬁndings consistent | Was there consistency between the data presented and the ﬁndings? | Yes, throughout |
| 31. Clarity of major themes | Were major themes clearly presented in the ﬁndings? | pp.8-23, Additional File 5 |
| 32. Clarity of minor themes | Is there a description of diverse cases or discussion of minor themes? | Additional File 5 |

Developed from:

Tong A, Sainsbury P, Craig J. Consolidated criteria for reporting qualitative research (COREQ): a 32-item checklist for interviews and focus groups. *International Journal for Quality in Health Care*. 2007. Volume 19, Number 6: pp. 349 – 357

**Personal Characteristics:**

Dr Susan Jones (Female). Research Fellow, with 15 years’ experience of conducting qualitative evaluations and research in public health.

Dr Tamara Brown (Female). Associate Professor of Obesity, with 5 years’ experience of focus groups and 20 years of experience of conducting research in weight management.

Pat Watson (Female). Research Fellow, with over 15 years’ experience of conducting qualitative evaluations and research in public health.

Professor Louisa Ells (Female). Professor of Obesity with a specialist interest in multi-disciplinary, cross-sector applied obesity research, with extensive experience of leading programme evaluations.

Dr Catherine Homer (Female). Associate Professor of Obesity and Public Health with experience working in academia and extensive experience working in public health.

Charlotte Freeman (Female). Project Research Assistant with experience of evaluating interventions in academia and experience of working in public health.

Dr Chirag Bakhai (Male). General Practitioner in primary care with a special interest in diabetes management. Chirag is a clinical lead on the Oversight group for the Re:Mission Study and a primary care advisor to the National Diabetes Programme for NHS England and NHS Improvement.
