# Supplementary material for: Commercial provider staff experiences of the NHS low calorie diet programme pilot: a qualitative exploration of key barriers and facilitators
Source: BMC Health Serv Res. 2024 Jan 10;24:53. doi: 10.1186/s12913-023-10501-y (PMC10782528; doi:10.1186/s12913-023-10501-y)
Supplement: Supplementary file 3 — Supplementary Material 3: Additional File 3 provides information on NPT and the focus group question framework [file 12913_2023_10501_MOESM3_ESM.docx]

# Additional File 3

## Normalisation Process Theory

NPT is a mid-range theory that has become popular in healthcare research, especially for exploring service organisation and intervention delivery (May & Finch, 2009). It has four mechanisms: coherence (sense-making), cognitive participation (engagement and participation), collective action (activation/workability) and reflexive monitoring (appraisal).

NPT has the advantage that it identifies the mechanisms / active ingredients required for an intervention to become part of routine care. Consequently, it is also able to show where the facilitators and barriers in the implementation process are found.

The data collection tool used with staff working in provider organisations was based on the NPT constructs. We were also interested to find out about equity and person-centredness. The schedule below is the generic version which was adapted to different organisations and staff roles as necessary.

## Service providers focus group schedule – generic frontline delivery staff

**Professional context**

1. **What are your job titles?**

- [Interviewer to check job titles in advance and confirm with the group]
- Are you the delivery staff for the LCD programme? In which locality/localities? [Do they live in the locality where they deliver the programme]?
- Please could you summarise your role for me?
- Which mode(s) of delivery do you offer? [Confirm with the group]

## COHERENCE – MEANING/SENSE MAKING

**Initial planning**

1. **What do you see as the aim of the programme?**

- What benefits are expected for service users?
- How do you think these benefits will be achieved?
- How does the programme differ from previous / other programmes (to manage weight and/or diabetes)?

1. **What is your understanding of how implementation was planned in your organisation?**

- What was your role in setting up the programme?

## COGNITIVE PARTICIPATION – COMMITMENT/BUY-IN

**Staff training**

1. **Did you receive appropriate training to be able to deliver the LCD programme?**

**If yes:**

- Was the purpose of the training clear?
- How useful has the training manual been?
- Did the content of the training make sense to you?
- Did behaviour change theory featured at all, in the training?
- How well has the training prepared you to deliver the programme?
- Did you/other trainees raise any issues/concerns with delivering the programme?

**If no:**

- Why was training not undertaken?
- Would training have supported delivery in any way?

**Communication**

1. **How easy to use are the materials?**

- How do your service users find the materials? (Do they reflect their lives?)
- How does the ease of use of the materials affect your engagement in the programme?
- How does the ease of use of the materials affect their (service users) engagement in the programme?

1. **How effective is the communication within the organisation in supporting the service user journey?**

- Do you give any feedback? Who to? What about?
- What feedback do you receive? E.g. relating to a) individual service users and b) the programme?

## COLLECTIVE ACTION – ACTIVATION/EFFORT

**Workability**

1. **Can you explain what happened when the programme was implemented?**

- Did it run according to plan?
- How well, in practice, were you able to adhere to the standard set out in the training?
- Were any barriers to provision/delivery (with fidelity) identified?
- Were any facilitators to provision/delivery (with fidelity) identified?
- Did integrating referral pathways into existing local care pathways raise any issues?
- In your opinion, how well were service users able to adhere to the programme?
- Was there any evidence of working through issues by a) staff and b) service users; active learning and readjustment of plans?
- How about now? Is the programme running as planned?

1. **Can you explain how you were/are kept up to date with any changes by your organisation?**

- During the setting up period?
- Once the programme was up and running?
- What about changes related to Covid-19, how were/are they communicated between you, NHS staff and your senior managers, so you were/are able to tell service users about them?

## ADDITIONAL TOPIC

**Equity**

1. **To what extent do you think the programme works for all eligible patients?**

- Do you think all the groups you would expect to see are on the programme? Any missing?
- Do all groups engage and participate to the same extent?
- Are there any particular groups who drop-out?
- Have you worked in this locality before?
- Are you aware of the characteristics of the locality/ population? E.g. Amenities, demographics, eligible groups living in the locality.

## ADDITIONAL TOPIC

**Person-centred**

1. **How well does tailoring the programme to the individual’s needs work across the different communities, groups and cultures on the programme?**

- In what ways has the programme been designed to be person-centred? i.e. to take into account personal characteristics, circumstances, beliefs, norms etc.

## REFLEXIVE MONITORING – REFLECTION/COMPREHENSION

**Sustainability**

1. **What do you think are the factors that make this programme work well?**
2. **And what are the barriers to it working well?**

- To what extent have health organisations embraced the programme?
- What about service users, to what extent have they bought into the programme?
- How have you been tailoring it to different contexts and populations?
- How has feedback informed changes?
- In what way(s) can you see the programme has/has not been integrated into standard services?
- What do you think the future holds for this programme?

**REFERENCE**

May C, and Finch T. Implementing, Embedding, and Integrating Practices: An Outline of Normalization Process Theory. Sociology, 2009; 43(3): 535–554.
