# Supplementary material for: Commercial provider staff experiences of the NHS low calorie diet programme pilot: a qualitative exploration of key barriers and facilitators
Source: BMC Health Serv Res. 2024 Jan 10;24:53. doi: 10.1186/s12913-023-10501-y (PMC10782528; doi:10.1186/s12913-023-10501-y)
Supplement: Supplementary file 4 — Supplementary Material 4: Additional File 4 provides the codebook and an example of data linkage [file 12913_2023_10501_MOESM4_ESM.docx]

# Additional File 4

NHS LCD Evaluation Provider Focus Groups

Nodes\\CODEBOOK 5\\Deductive and inductive coding v5

| Name | Description |
| --- | --- |
| 1 CONTEXT |  |
| IMPACT OF C19 | The impact of covid on the pilot of the LCD programme. |
| Dropout | Effect on dropout that is due to covid, including reasons and examples. |
| Launching the programme | The impact of covid-19 on launching the programme |
| Online delivery | How covid-19 has impacted on delivery, moving from planned in-person to online or virtual delivery |
| Primary care | The impact of covid-19 on the relationship with primary care and referrals from primary care |
| Macro level | National/covid-19 |
| Medium level | Provider/organisation/team |
| Micro level | Individual service users and deliverers, we are talking about the facts rather than individual level perceptions |
| 2 MECHANISMS | Nodes based on NPT core concepts with free coding to inductive sub nodes |
| 1 Coherence or making sense of it | The process of sense-making and understanding that individuals and organisations have to go through in order to promote or inhibit the routine embedding of a practice to its users. These processes are energised by investments of meaning made by participants. How people understand and make sense of a practice with an emphasis on understanding and conceptualisation. |
| Aim and purpose of the LCD programme (D) | Defines the aim and purpose of the programme, Differentiates this programme from others. |
| Differentiation | How the new LCD programme differed from existing practice and programmes. |
| Difference from other programmes |  |
| High level of support differentiates the LCD programme |  |
| Intensity differentiates the LCD programme |  |
| TDR differentiates LCD programme |  |
| Individually making sense of the programme | Making sense of personal versions of tasks for yourself as a team member - individual specification (NPT component) |
| SU measurements |  |
| Working out the benefits of the programme yourself (I) | Internalisation - working out for yourself what the benefits are of the LCD programme. |
| Working out the rationale for the programme for yourself (IS) | Internal specification |
| Location of staff and delivery site |  |
| Making sense of the programme across providers and referrers | Shared understanding of aims, objectives and expected benefits of the LCD programme between providers and referrers. Communal specification (NPT component) |
| Information from providers to referrers | Type and frequency of information given by service providers, helpfulness of this information. Includes discussion of the need for more information on patient progress that would be helpful for referrers. |
| Information given supported making a referral to programme | Information given about the programme was helpful to referrers, online resources useful, it prepared them for making referrals, |
| Suitability of referrals (IS) | Making sense of who is suitable to go on the programme |
| 2 Cognitive Participation or working out participation | The process that individuals and organisations have to go through in order to enrol individuals to engage with the new practice. These processes are energised by investments of commitment made by participants. How people engage and participate with an emphasis on legitimation and buy in. |
| Communication | Internal and external communication by the provider |
| Coaches | Internal communication with and between coaches delivering the programme |
| Feedback | Feedback from service users; communication to coaches and internally between staff within the provider organisation. |
| Middle and Senior management team (ops) | Internal communication between the senior managers, the middle managers (contract liaison officers) and the frontline staff delivering the LCD programme. |
| Patient Support Team | Communication with the Patient Support Team, or role, that bridges between the patient and the LCD programme provider. |
| Primary care | Communicating with primary care, including GPs, at the implementation/set up stage |
| Content and delivery of training | What was covered in the training for referrers, how was it delivered and the benefits and limitations of online delivery. How resources are used in practice – slides and guidance booklet. Discussion of how to deliver training from perspective of clinical lead, |
| Enroling and supporting delivery staff | Process for brining delivery staff on board with the idea of the new LCD programme. |
| Gaps in training | Any area where training was insufficient |
| Mobilisation | Enrolling individuals into the idea of the LCD programme; Activating individuals to sustain involvement; Initiating individuals into driving the LCD programme |
| Role of the Patient Support Team | How the Patient Support Team fits into staff structures and relates to the patients |
| 3 Collective Action or doing it | The work that individuals and organisations have to do to enact the new practice. These processes are energised by investments of effort made by participants. The work required and resources needed to support. Emphasis on resources, training, divisions of labour, confidence and expertise, workability. |
| Barriers to collective action | This is an overarching node to capture all the barriers to implementing and running the LCD programme. |
| Barriers that are service user related | Barriers to the implementation and running of the LCD programme that are directly related to the service users |
| Barriers to delivery of the programme (online) | Includes all system and process barriers to delivery of the LCD programme - also those raised by covid and going online rather than face2face; lack of barriers to delivery |
| Barriers to referring | Descriptions of factors that have prevented or impeded referrals to the LCD programme |
| Issues relating to deprescribing | Deprescribing as a barrier to referring onto the LCD programme e.g. for smaller practices without a pharmacist, or missing the requirement of programme to give patient information about medication changes, |
| Process factors | Barriers to referring onto the LCD programme relating to the standard processes of the intervention |
| Programme factors | Strict referral criteria presenting barriers to referral onto the LCD programme, patients previous experience of TDR products as a disincentive, group format creating delays (waiting for sufficient numbers), |
| Workload constraints | Perceptions of additional workload for referrer on making a referral, additional or sheer volume of demands being placed on staff, referral appears to be or is a time-consuming process, lack of capacity, |
| Perception of reasons for dropout | Providers’ perceptions of why service users drop out of the LCD programme |
| Facilitators to collective action | This is an overarching node to capture all the facilitators to implementing and running the LCD programme |
| Bridging role between funder, referrer and deliverer of programme | Management role to link between funder, referrer and frontline delivery of the programme: what it is, how it functions, what it adds. |
| Facilitators of delivering the programme (online) | Includes all system and process facilitators to delivery of the LCD programme - also those relating to covid and going online rather than face2face. |
| Resources for service users | Resources provided for service users that facilitated their engagement with the LCD programme |
| Systems | Systems factors that support and facilitate delivery of the LCD programme |
| Facilitators of referrals | Descriptions of factors that have facilitated referrals to the LCD programme |
| Facilitators that are service user related | Facilitators to the implementation and running of the LCD programme that are directly related to the service users. |
| Management of referrals and bookings | Description of process of receiving referrals and booking service users onto the programme after referrals are generated in their practice |
| Deprescribing | How medication changes are managed in the practice |
| Management of bookings by Patient Support Team | Description of what happens once the provider receives the referral from the GP practice and booking onto the programme begins |
| Opportunistic referrals | Discussion of opportunities for making referral - limitations and advantages this presents. Opportunity at annual review, on new diagnosis of T2D. Who might be missing? Secondary care route, self-referral. |
| Referral allocation within locality | Capping of numbers and how this affected referrals. How referring changed when allocation removed, |
| Supporting GP practice staff | Description of how providers have supported GP practice staff in the referral and follow up process |
| 4 Reflexive Monitoring or reflecting on it | The informal and formal appraisal of a new practice once it is in use, in order to assess its advantages and disadvantages and which develops users’ comprehension of the effects of a practice. These processes are energised by investments in appraisal made by participants. How effects are appraised. Emphasis on appraising and monitoring. |
| Internal feedback | Examples of feedback among staff that is present or desired |
| Ways to improve referrals | Suggestions based on current practice and limitations of this. Includes marketing of programme, awareness of what’s involved in programme, focus more on Practice Nurses making referrals as they have more holistic perspective on patient, giving reassurance about adverse events and medication management, reassurance about workload remaining manageable – having searches set up, incentives for referrals, practice champions, including those diagnosed for longer time, |
| Wider system level changes to support programme | Discussion of changes needed across wider health economy for programme to be sustainable – creation of a referral hub, referring patient to right service for them, support for practices |
| Learning from implementation process | Reflecting on what has been learnt from the pilot |
| Motivation of individual | Examples of individual motivation for the programme, associated outcomes and implications of differing levels of motivation |
| Programme becomes part of normal practice | Programme becomes part of business as usual, familiarity makes it easier, requirements of referral can be managed efficiently, no additional demands on practitioners |
| Programme seen as a good thing | Positive view of programme, positive impacts seen to date, success stories, |
| Reviewing LCD programme | Ideas for improving the programme, often using feedback from staff and service users. |
| Ways to improve equity of programme | Ideas for improving equity in delivery of the programme e,g, use community languages, digital inclusion programmes, |
| Ways to improve products | Ideas for improving the acceptability of TDR products with a view to improving outcomes |
| 3 EQUITY | Health equity (the state in which people have a fair and just opportunity, irrespective of their social position, to attain their full health and welling from social conditions that seek to promote and support good health Williams O, Coen SE, Gibson K. Comment on: "Equity in Physical Activity: A Misguided Goal". Sports Medicine. 2019;49:637-9.). How equitable is the pilot LCD programme? Who is missing among the service users, who is represented? What has increased/decreased equity? |
| Cultural competence of the programme | Cultural competence in relation to programme delivery including appropriateness of materials to social norms of minority cultures |
| Differences between areas | Noting different requirements and different provision between different geographical locations in relation to equity |
| Digital engagement | Considerations of those excluded/included by digital delivery including online delivery, use and access to technology and support materials |
| Digital pathway | References to the digital pathway. Telephone or video call are considered different pathways, while neither being face 2 face nor online individual sessions with online resources via the app. Reflecting on whether the telephone coaching pathway impacts on equity. |
| Equity of the referral process | Examples of how equity has been managed in the referral process |
| Language as a barrier | Where English is not a first language, or it is not well understood |
| Product related equity | Examples of ways equity has been increased in relation to TDR products |
| Sample representativeness | Examples of equity issues around referral and retention processes |
| Socioeconomic equity | Examples of diverse levels of wealth and poverty and how the programme seeks to be available to all regardless of socioeconomic factors |
| 4 PERSON CENTREDNESS | To what extent has the pilot LCD programme been implemented and delivered in a person-centred manner? |
| Individual calls to service users outside group session | Data about the individual telephone calls that coaches have with service users who attend group sessions, outside the main session e.g. beforehand to report on measurements such as weight. |
| Integrating programme, person's needs and healthcare systems | Integrating the programme with the needs of the individual and the wider healthcare systems, to include medical monitoring and medicine management, multidisciplinary working, increased individual contact for co-morbidities, extra language support |
| Peer support between service users | Any form of support that the service users give to one another. Examples include group support through the chat function, talking in sessions, WhatsApp outside of sessions. |
| Programme - making it work for the individual | Description of how the programme design and delivery is person centred (or not) to include personal action plan, goals, choices, problem-solving, tailoring to individual requirements |
| Sessions in Urdu and Hindi | Provision of group session with Urdu/Hindi speaker |
| Staff and service user relationships | Personalisation through the conduct of staff/service user relationships within the LCD programme |
| 5 COSTS AND RESOURCES | [For health economist] What cost and resource implications have been indirectly or directly identified? |
| Cultural adaptations | Examples of cultural adaptations to the LCD programme |
| Effectiveness | Anecdotal examples of effectiveness |
| Medical management and liaison with primary care | Examples of medical management and liaison with primary care that might have economic implications |
| Online resources | Examples that might have economic implications e.g. changes required for online delivery related to system usability and admin system for coaches, revising supporting materials to online, continuing to produce paper materials |
| Product | Examples relating to the TDR products that might have economic implications e.g. product provider changes, product delivery (parcel firm) and provision of samples |
| Referral costs | Examples within the referral process that might have economic implications e.g. liaison work with various health professionals and various routes of referral |
| Systems | Examples that might have economic implications, including computer link up between practices and centrally |
| Travel | Examples that might have economic implications e.g. costs of travel expenses and time for coaches and providers to deliver LCD programme |

## Details of adapting the codebook from the analysis of referrer staff data

TB and SJ carefully considered the process of analysis recently undertaken by CF for the referrer interview data as part of the Re:Mission study (Ells *et al*., 2021). CF had initially open coded the referrer data and then coded inductively and deductively, by using the main codes from the open coding and reorganising them, using the Normalisation Process Theory (NPT) (May & Finch, 2009) and the context, mechanism, outcome framework of Realist Evaluation (Pawson & Tilley, 2004) as codes. Main codes included Context; Mechanisms (sub-codes of the 4 NPT concepts of coherence, cognitive participation, collective action, reflexive monitoring); Outcomes; Equity; and Person-centredness.

TB and SJ open coded one provider interview transcript to the open coding codebook from the referrer data. TB and SB coded another transcript to the inductive and deductive codebook from the referrer data, amended to remove the ‘outcomes’ node, to see which codebook provided a ‘better fit’. The inductive and deductive codebook was broadly appropriate and covered the key topics of the research questions: provider experience, barriers, facilitators and normalisation. Most differences in coding between TB and SB related to the *amount* of text coded around the key issue rather than differences in allocating codes. TB and SJ agreed that the referrer sub-codes under ‘context’ were not relevant to the provider data, so these were deleted. Instead, macro, medium and micro levels were added.

## Example of linkage between data and findings

# Extract from concise summary statement for Theme – Cognitive Participation; sub-node Enrolling and Supporting Delivery (see Additional File 5 for full summary statement)

| **Tree node** | **Summary statement** |
| --- | --- |
| **CP.3.3 Cognitive Participation/enrolling and supporting delivery** | Recognition of importance of well-structured training to enrol the coaches into the provider’s systems and programme content |

Analysis:

The importance of well-structured training to enrol the coach into the provider’s systems and programme content was recognised:

“[PS5] organised some training for me and really, the, the development plan, the training, the organisation and how it was set up was really well structured, well organised, sequential.” (PFG02, PS06)

Well-structured training prepared the coach to support the service user well:

“… it really set us up to win really, so that when you had your first ones, although you can never really get ready for your first one 'cause you don't know what your first one is going to be like, I think it's probably the best prepared I’ve ever been for [sic] a training session.” (PFG02, PS06)

In depth training in the programme content prepared the coach to support the service user well:

“I think all those [elements of the programme] are covered to a really good depth in terms of the, the training cycle and that means that when you do go out into the field you feel well prepared and organised.” (PFG02, PS06)

REFERENCES

Ells L, Radley, D., Homer, C. A coproduced mixed method evaluation of the NHS England Low-Calorie Diet implementation pilot. Protocol. 2021 12th March 2021.

May C, and Finch T. Implementing, Embedding, and Integrating Practices: An Outline of Normalization Process Theory. Sociology, 2009; 43(3): 535–554.

Pawson, R. & Tilley, N. Realistic Evaluation. London, British Cabinet Office, 2004.

https://www.alnap.org/system/files/content/resource/files/main/pawson---tilley-%282004%29-realist-evaluation.pdf
