# Supplementary material for: Commercial provider staff experiences of the NHS low calorie diet programme pilot: a qualitative exploration of key barriers and facilitators
Source: BMC Health Serv Res. 2024 Jan 10;24:53. doi: 10.1186/s12913-023-10501-y (PMC10782528; doi:10.1186/s12913-023-10501-y)
Supplement: Supplementary file 5 — Supplementary Material 5: Additional File 5 provides further findings and quotations from the data [file 12913_2023_10501_MOESM5_ESM.docx]

# Additional File 5

## Analytical themes

| **Number** | **Analytical theme** |
| --- | --- |
| 1 | Perceptions that promote programme outcomes |
| 2 | Perceptions that are barriers to programme outcomes |
| 3 | Personal characteristics and circumstances of SUs that are barriers to programme outcomes |
| 4 | Personal characteristics and circumstances of SUs that promote programme outcomes |
| 5 | Systems that promote programme outcomes |
| 6 | Systems that are barriers to programme outcomes |
| 7 | Structures that promote programme outcomes |
| 8 | Structures that are barriers to programme outcomes |
| 9 | Structures that promote normalisation |
| 10 | Structures that are barriers to normalisation |
| 11 | Factors that promote normalisation |
| 12 | Factors that are barriers to normalisation |
| 13 | Factors that promote equity |

### Definitions of words used in analytical themes

Programme outcomes = the outcomes identified in the NHSE specification

Structures = frameworks/plans within organisations e.g. the division of roles and responsibilities, the way teams are organised, the plan to achieve programme outcomes

Systems = processes within organisations e.g. IT systems, administrative activities

Factors = items within the structures and/or systems that affect the programme outcomes

Promote = items within the structures and/or systems that aid reaching the programme outcomes; includes facilitation

Barriers = items within the structures and/or systems that limit or obstruct reaching the programme outcomes

Normalisation = the potential of an intervention for ‘workability’, ‘fit’ and integration into current practice (May *et al.* 2007)

## Summary Statements and Data Linkage Tables

## Coherence

### **Table: Concise summary statements for Theme – Coherence**

| **Tree nodes** | **Summary statement** | **Code** |
| --- | --- | --- |
| **C.1. Coherence/Aim and purpose of the LCD programme** | Debate by staff over whether the primary aim is diabetes remission or weight loss; included factors which contribute to meeting the primary aim. | C.1.1 |
| **C.2 Coherence/Aim and purpose for the service user (SU) – coding notes** | SUs identified diabetes remission as the aim, closely followed by weight loss; included sustainable changes. | C.2.1 |
| **C.3. 1 Coherence/Differentiation**  **Difference from other programmes** | For staff – LCD more complex and onerous. | C.3. 1.1 |
|  | For SUs – LCD more holistic and sustainable. | C.3. 1.2 |
|  | SUs had greater programme readiness, higher commitment and engagement. | C.3. 1.3 |
|  | Delivery models were more varied, with one-to-one and an online learning via an app, in addition to group sessions (depending on site). | C.3. 1.4 |
|  | TDR phase and products was a key difference, with weight loss reported as quicker in the early stages. | C.3.1.5 |
|  | Long-term support and follow up for one year. | C.3.1.6 |
| **C.3.2 Coherence/Differentiation**  **High level of support** | Extended length of time SUs receive support. | C.3.2 |
| **C.3.3 Coherence/Differentiation**  **Intensity** | More intense for SUs, especially at the start. | C.3.3.1 |
|  | More support offered by staff, especially at the start. | C.3.3.2 |
|  | More challenging for SUs to fit into daily life. | C.3.3.3 |
|  | More likely to be sustainable for the SU. | C.3.3.4 |
| **C.3.4**  **Coherence/Differentiation**  **TDR** | Complete removal of food for the first 12 weeks, replaced by specific products. | C.3.4.1 |
|  | TDR gives a pause and an opportunity for a reset. | C.3.4.2 |
|  | TDR is a way to create a new start. | C.3.4.3 |
|  | TDR often leads to dramatic and motivational weight loss. | C.3.4.4 |
| **C.4.1 Coherence/ Individually making sense of the programme/**  **SU measurements** | Self-monitoring was motivational and reduced work for primary care. | C.4.1.1 |
|  | Key measures were reductions in weight, blood glucose and medication. | C.4.1.2 |
|  | Preparing for long-term health. | C.4.1.3 |
| **C4.2 Coherence/** **Individually making sense of the programme/**  **Working out the benefits of the LCD programme yourself** | To recruit GPs to refer suitable patients to promote diabetes remission. | C.4.2.1 |
|  | To reduce GP workload and to offer a joined-up service. | C.4.2.2 |
|  | Professionals observed SUs physical and mental health benefitting. | C.4.2.3 |
|  | Variety of modes of delivery appeared of benefit. | C.4.2.4 |
| **C.4.3 Coherence/** **Individually making sense of the programme/**  **Working out the rationale for the programme for yourself** | Staff role was to support service users to reduce their blood glucose, to bring their diabetes into remission, and to tailor the session to the individual. | C.4.3.1 |
|  | The programme used reflection, education and empowerment. | C.4.3.2 |
|  | Standardised materials provided to ensure all were given same information. | C.4.3.3 |
|  | SUs were observed to experience fast weight loss and health improvement. | C.4.3.4 |
|  | Need to manage the reintroduction of food to maximise sustainable behaviour change. | C.4.3.5 |
| **C.5.1 Coherence/ Making sense of the programme across providers to referrers/**  **Information from providers to referrers** | Provider created new process, resources and training materials for primary care. | C.5.1 |
| **C.5.2 Coherence/ Making sense of the programme across providers to referrers/**  **Information given supported making a referral to programme** | GPs needed information that made it clear which patients would benefit most from this programme, in the context of several programmes to choose from. | C.5.2 |
| **C.5.3 Coherence/ Making sense of the programme across providers to referrers/**  **Suitability of referrals** | Suitable referrals were a key issue for success. | C.5.3.1 |
|  | Examples of suitable referrals who did well and unsuitable referrals who did not. | C.5.3.2 |

### Linking NPT construct COHERENCE with descriptive and analytical themes

*To be read in conjunction with the summary statement for COHERENCE*

| **NPT construct** | **Descriptive theme** | **Analytical theme** | **Code** |
| --- | --- | --- | --- |
| **C.1. Coherence** | **Aim and purpose of the LCD programme for provider staff** | Perceptions that are barriers to programme outcomes | C.1.1 |
| **C.2 Coherence** | **Aim and purpose for the client** | Perceptions that are barriers to programme outcomes | C.2.1 |
| **C.3. 1 Coherence** | **Differentiation/Difference from other programmes** | Perceptions that are barriers to programme outcomes | C.3. 1.1 |
|  |  |  | C.3. 1.2 |
|  |  |  | C.3. 1.3 |
|  |  |  | C.3. 1.4 |
|  |  |  | C.3.1.5 |
|  |  |  | C.3.1.6 |
| **C.3.2 Coherence** | **Differentiation/High level of support** | Perceptions that promote programme outcomes | C.3.2 |
| **C.3.3 Coherence** | **Differentiation/Intensity** | Perceptions that promote programme outcomes | C.3.3.1 |
|  |  |  | C.3.3.2 |
|  |  |  | C.3.3.3 |
|  |  |  | C.3.3.4 |
| **C.3.4 Coherence** | **Differentiation/TDR** | Perceptions that promote programme outcomes | C.3.4.1 |
|  |  |  | C.3.4.2 |
|  |  |  | C.3.4.3 |
|  |  |  | C.3.4.4 |
| **C.4.1 Coherence** | **Individually making sense of the programme/SU measurements** | Perceptions that promote programme outcomes | C.4.1.1 |
|  |  |  | C.4.1.2 |
|  |  |  | C.4.1.3 |
| **C4.2 Coherence** | **Individually making sense of the programme/Working out the benefits of the LCD programme yourself** | Perceptions that promote programme outcomes | C.4.2.1 |
|  |  |  | C.4.2.2 |
|  |  |  | C.4.2.3 |
|  |  |  | C.4.2.4 |
| **C.4.3 Coherence** | **Individually making sense of the programme/Working out the rationale for the programme for yourself** | Perceptions that promote programme outcomes | C.4.3.1 |
|  |  |  | C.4.3.2 |
|  |  |  | C.4.3.3 |
|  |  |  | C.4.3.4 |
|  |  |  | C.4.3.5 |
| **C.5.1 Coherence** | **Making sense of the programme across providers to referrers/Information from providers to referrers** | Perceptions that promote programme outcomes | C.5.1 |
| **C.5.2 Coherence** | **Making sense of the programme across providers to referrers/Information given supported making a referral to programme** | Perceptions that promote programme outcomes | C.5.2 |
| **C.5.3 Coherence** | **Making sense of the programme across providers to referrers/Suitability of referrals** | Perceptions that promote programme outcomes  Perceptions that are barriers to programme outcomes | C.5.3.1 |
|  |  |  | C.5.3.2 |

## Cognitive Participation

### **Table: Concise summary statements for Theme – Cognitive participation – sub-node Communication**

| **Tree nodes** | **Summary statement** |  | **Code** |
| --- | --- | --- | --- |
| **CP.1. Cognitive participation/Communication** | Internal and external communication by the providers to all the stakeholders |  | CP.1 |
|  | **CP.1.1**  **Coaches**  Communication to and from the coaches to other stakeholders |  | CP.1.1 |
|  |  | **CP.1.1.1**  **Communication with service users** Communication to and from the coaches to service users | CP.1.1.1 |
|  |  | Importance of programme length | CP.1.1.2 |
|  |  | Importance of initial contact | CP.1.1.3 |
|  |  | **CP.1.1.2**  **Internal communication**  Communication to and from the coaches within the provider organisation | CP.1.1.2 |
|  |  | Systems varied across providers | CP.1.1.2.1 |
|  |  | Email update on statistical data – planned/expected | CP.1.1.2.2 |
|  |  | Email update on statistical data – unplanned/unexpected | CP.1.1.2.3 |
|  |  | Regular ‘keep-in-touch’ call (KIT calls) from managers | CP.1.1.2.4 |
|  |  | Online chat groups, including peers and managers | CP.1.1.2.5 |
|  |  | Peer-peer, peer-manager emails | CP.1.1.2.6 |
|  |  | Monthly drop-in organised by managers | CP.1.1.2.7 |
|  |  | Quarterly team meeting/training day organised by managers | CP.1.1.2.8 |
|  |  | Process to escalate queries/concerns to senior managers/ MDT | CP.1.1.2.9 |
|  |  | Online tracker systems  “Remote registers” where reference information is stored | CP.1.1.2.10 |
|  | **CP.1.2**  **Feedback** |  | CP.1.2 |
|  | Feedback systems used by provider organisations   - Internal - Routine data collection (quantitative) - Internal - Performance of coaches (observational) - External - From SUs/ other stakeholders to provider (qualitative) - External – From provider to SUs/other stakeholders |  | CP.1.2.1 |
|  | Quantitative data analysed and circulated internally and to stakeholders |  | CP.1.2.2 |
|  | Communication loops present – for negative/positive feedback; health incidents; SU queries |  | CP.1.2.3 |
|  | Feedback used for   - Service improvement - Maintaining quality - Encouragement - Professional development |  | CP.1.2.4 |
|  | Digital feedback provided for SUs on digital mode |  |  |
|  | **CP.1.3**  **Patient Support Team (PST)** |  | CP.1.3 |
|  | Communication to and from the PST within the provider organisation |  | CP.1.3.1 |
|  | Only one provider used this model |  | CP.1.3.2 |
|  | Initial point of access for SUs to contact the programme |  | CP.1.3.3 |
|  | Provided a thorough preparation for the SUs before they started the programme |  | CP.1.3.4 |
|  | Weekly meeting with middle managers |  | CP.1.3.5 |
|  | Peer-peer support |  | CP.1.3.6 |
|  | **CP.1.4**  **Primary care** |  | CP.1.4 |
|  | Communication between the provider and primary care/GP practices |  | CP.1.4.1 |
|  | Fundamental to all referrals |  | CP.1.4.2 |
|  | Work by provider needed to bring GP practices on board  ‘finding the chinks in that armour’ to gain access to GPs |  | CP.1.4.3 |
|  | Buy-in by GP practices involved awareness raising and discussion |  | CP.1.4.4 |
|  | Correct identification by GPs of eligible patients was paramount for successful patient outcomes |  | CP.1.4.5 |
|  | Variable depth of information provided by the GP to their patient about the programme |  | CP.1.4.6 |
|  | **CP.1.5**  **Middle and senior management team** |  | CP.1.5 |
|  | Communication within the management and out to external stakeholders |  | CP.1.5.1 |
|  | Structures varied across providers |  | CP.1.5.2 |
|  | Bridging role   - Maintain the contract - Deliver effectively - Priority setting - Creating solutions |  | CP.1.5.3 |
|  | Structured reporting mechanisms |  | CP.1.5.4 |
|  | Unstructured reporting mechanisms |  | CP.1.5.5 |
|  | Attributes of communication   - Collaborative - Cross-departmental |  | CP.1.5.6 |

### Linking NPT construct COGNITIVE PARTICIPATION with descriptive and analytical themes for COMMUNICATION

*To be read in conjunction with the summary statement for COGNITIVE PARTICIPATION*

| **NPT construct** | **Descriptive theme** | **Analytical theme** | **Code** |
| --- | --- | --- | --- |
| **CP.1. Cognitive participation/Communication** | **CP.1.1**  **Coaches**  Communication to and from the coaches to other stakeholders | Structures that promote programme outcomes | CP.1.1 |
|  |  |  | CP.1.1.1 |
|  |  |  | CP.1.1.2 |
|  |  |  | CP.1.1.3 |
|  |  |  | CP.1.1.2 |
|  |  |  | CP.1.1.2.1 |
|  |  |  | CP.1.1.2.2 |
|  |  |  | CP.1.1.2.3 |
|  |  |  | CP.1.1.2.4 |
|  |  |  | CP.1.1.2.5 |
|  |  |  | CP.1.1.2.6 |
|  |  |  | CP.1.1.2.7 |
|  |  |  | CP.1.1.2.8 |
|  |  |  | CP.1.1.2.9 |
|  |  |  | CP.1.1.2.10 |
|  | **CP.1.2**  **Feedback**  Feedback systems used by provider organisations | Structures that promote programme outcomes | CP.1.2 |
|  |  |  | CP.1.2.1 |
|  |  |  | CP.1.2.2 |
|  |  |  | CP.1.2.3 |
|  |  |  | CP.1.2.4 |
|  | **CP.1.3**  **Patient Support Team (PST)** | Structures that promote programme outcomes | CP.1.3 |
|  |  |  | CP.1.3.1 |
|  |  |  | CP.1.3.2 |
|  |  |  | CP.1.3.3 |
|  |  |  | CP.1.3.4 |
|  |  |  | CP.1.3.5 |
|  |  |  | CP.1.3.6 |
|  | **CP.1.4**  **Primary care** | Structures that are barriers to programme outcomes  Structures that promote programme outcomes | CP.1.4 |
|  |  |  | CP.1.4.1 |
|  |  |  | CP.1.4.2 |
|  |  |  | CP.1.4.3 |
|  |  |  | CP.1.4.4 |
|  |  |  | CP.1.4.5 |
|  |  |  | CP.1.4.6 |
|  | **CP.1.5**  **Middle and senior management team** | Structures that are barriers to programme outcomes  Structures that promote programme outcomes | CP.1.5 |
|  |  |  | CP.1.5.1 |
|  |  |  | CP.1.5.2 |
|  |  |  | CP.1.5.3 |
|  |  |  | CP.1.5.4 |
|  |  |  | CP.1.5.5 |
|  |  |  | CP.1.5.6 |

### **Table: Concise summary statements for Theme – Cognitive Participation - sub-node Content and Delivery of Training**

| **Tree nodes** | **Sub code** | **Summary statement** | **Code** |
| --- | --- | --- | --- |
| **CP 3.2 Cognitive participation/Content & delivery of training** | **CP.3.2.1**  **Training content** | The structure of the programme was prescribed by the NHS to the providers (20 sessions, 3 phases) and so the training of the coaches focussed on the structure of the programme. | CP.3.2.1.1 |
|  |  | Training includes a development plan, content of the programme, and about the organisation. This is called ‘standard onboarding’. | CP.3.2.1.2 |
|  |  | Training includes awareness of accessibility of digital format and online delivery. | CP.3.2.1.3 |
|  |  | Organised training **content** includes: Eatwell Guide; Portion control; Food diaries; Reviews and ‘smart reviews’; Meal planning; Overall wellbeing including mindfulness | CP.3.2.1.4 |
|  |  | Different ‘types’ of training included: clinical skills training, behaviour change techniques (COM-B, stages of change, motivational interviewing), programme content, administration/procedures, monitoring the contract through KPIs, App training, software training. | CP.3.2.1.5 |
|  |  | Behaviour change is an extensive part of the **content** of the training. | CP.3.2.1.6 |
|  |  | Group and one-to-one delivery model training content was the same, the difference is in how the coaches tailor the content to the person. | CP.3.2.1.7 |
|  | **CP.3.2.2**  **Training changed as programme became more established** | Training differed during and after initial set-up phase; during initial set-up, coaches and trainers discussed where the flexibility within the structure was and providers had to keep checking with the NHS if they were doing it correctly. | CP.3.2.2.1 |
|  |  | After initial set-up phase, there was a move from coaches figuring it out for themselves and the programme being new and a ‘novel’ approach, towards coaches knowing what to expect and receiving more organised training. | CP.3.2.2.2 |
|  | **CP.3.2.3**  **Training changed to adapt to changed mode of delivery** | The programme was planned to be delivered face-to-face, so providers had to work out how to change resources into digital resources or postal. and one-to-one models – this is where coaches learned how to tailor the programme according to delivery model. | CP.3.2.3.1 |
|  | **CP.3.2.4**  **Training adapted to staff role** | Different ‘intensity’ of behaviour change training dependent on role, enables all staff to have an awareness and appreciation of where they fit within the team and role of others. | CP.3.2.4.1 |
|  |  | Training provides role scope. Training emphasises what is within and outside of the coaches’ role. | CP.3.2.4.2 |
|  | **CP.3.2.5**  **Training methods** | (Induction) Training includes ‘learning on the job’ through sitting in on other coaches’ sessions, listening in to booking calls, and shadowing experienced staff. | CP.3.2.5.1 |
|  |  | Training is interactive and includes role play. | CP.3.2.5.2 |
|  |  | Training includes extensive motivational interviewing, not just for the coaches to promote behaviour change in SUs but for Patient Pathway Co-ordinators to get people enrolled onto the programme. | CP.3.2.5.3 |
|  |  | There is ‘basic’ training, then training to help tailor delivery and learning how to apply the training, which includes ‘competency checks’, ‘supervision sessions’ and ‘additional training’. | CP.3.2.5.4 |
|  |  | Supervision sessions (by regional trainers) are separate for group mode. | CP.3.2.5.5 |
|  |  | Continuous learning through supervision sessions is highly valued by the providers. | CP.3.2.5.6 |
|  |  | Competency checks ensure continuous learning by ensuring forms are completed correctly, and correct procedures are followed and delivered appropriately. | CP.3.2.5.7 |
|  | **CP.3.2.6**  **Staff perceptions of receiving training** | Digital delivery is viewed as easier by the coaches because it is straightforward. SUs do their learning on the App so the coach training focusses on the motivational interviewing calls with SUs. | CP.3.2.6.1 |
|  |  | Training is viewed as overwhelmingly positive. | CP.3.2.6.2 |
|  |  | Trainer is role specific. Training of nutritional content is led by the dietitian. Physical activity lead does the physical activity session training. Clinical psychologist leads the behaviour change element of the training. | CP.3.2.6.3 |
|  | **CP.3.2.7**  **Normalisation of programme through positive experience** | Coaches put their learning of behaviour change techniques into action and see the positive difference it makes to their SUs’ journey. | CP.3.2.7.1 |
|  |  | Providers value the support aspect of the training and they see this support as an ongoing element beyond the training sessions. | CP.3.2.7.2 |
|  | **CP.3.2.8**  **Normalisation of programme through adaptation** | Training is responsive to need. | CP.3.2.8.1 |
|  |  | Continuous learning occurs through learning from each other and passing on training to others. | CP.3.2.8.2 |
|  |  | Training of **referrers** varies in intensity between the areas and the roll-out and is evolving with time. Project leads and diabetes clinical leads of each area influenced the content/delivery of the training of referrers, although the key parts remained the same. | CP.3.2.8.3 |
|  |  | Providers training themselves on service improvement. | CP.3.2.8.4 |
|  |  | The programme is a new clinical way of working with more clinical responsibility. | CP.3.2.8.5 |
|  | **CP.3.2.9**  **Training of referrers facilitates mobilisation** | Training of **referrers** was seen as an important part of the mobilisation phase, to raise awareness of the programme, to understand deprescribing, and how to refer. | CP.3.2.9.1 |
|  |  | One area stipulates they want **referrers** who have accessed the training in order for them to be eligible to refer. This training of referrers is available either ‘live’ or as a recording. | CP.3.2.9.2 |
|  | **CP.3.2.10**  **Challenges to be addressed in training** | Bringing in new people is **challenging** - having a really solid ‘onboarding’ structure for new staff is key. | CP.3.2.10.1 |
|  |  | Biggest **challenge** for coaches was moving from office-based to digital health. | CP.3.2.10.2 |
|  |  | **Challenge** of training people within a pilot adds extra pressure to the clinical team and in training people, with additional administration and monitoring and pressure of time and everything needing priority. | CP.3.2.10.3 |

### Linking NPT construct COGNITIVE PARTICIPATION with descriptive and analytical themes for CONTENT & DELIVERY OF TRAINING

*To be read in conjunction with the summary statement for COGNITIVE PARTICIPATION*

| **NPT construct** | **Descriptive theme** | **Analytical theme** | **Code** |
| --- | --- | --- | --- |
| **CP 3.2 Cognitive participation/Content & delivery of training** | **CP.3.2.1**  **Training content** | Structures that promote programme outcomes | CP.3.2.1.1 |
|  |  |  | CP.3.2.1.2 |
|  |  |  | CP.3.2.1.3 |
|  |  |  | CP.3.2.1.4 |
|  |  |  | CP.3.2.1.5 |
|  |  |  | CP.3.2.1.6 |
|  |  |  | CP.3.2.1.7 |
|  | **CP.3.2.2**  **Training changed as programme became more established** | Structures that promote programme outcomes | CP.3.2.2.1 |
|  |  |  | CP.3.2.2.2 |
|  | **CP.3.2.3**  **Training changed to adapt to changed mode of delivery** | Structures that promote programme outcomes | CP.3.2.3.1 |
|  | **CP.3.2.4**  **Training adapted to staff role** | Structures that promote programme outcomes | CP.3.2.4.1 |
|  |  |  | CP.3.2.4.2 |
|  | **CP.3.2.5**  **Training methods** | Structures that promote programme outcomes | CP.3.2.5.1 |
|  |  |  | CP.3.2.5.2 |
|  |  |  | CP.3.2.5.3 |
|  |  |  | CP.3.2.5.4 |
|  |  |  | CP.3.2.5.5 |
|  |  |  | CP.3.2.5.6 |
|  |  |  | CP.3.2.5.7 |
|  | **CP.3.2.6**  **Staff perceptions of receiving training** | Structures that promote programme outcomes | CP.3.2.6.1 |
|  |  |  | CP.3.2.6.2 |
|  |  |  | CP.3.2.6.3 |
|  | **CP.3.2.7**  **Normalisation of programme through positive experience** | Factors that promote normalisation | CP.3.2.7.1 |
|  |  |  | CP.3.2.7.2 |
|  | **CP.3.2.8**  **Normalisation of programme through adaptation** | Structures that promote normalisation | CP.3.2.8.1 |
|  |  |  | CP.3.2.8.2 |
|  |  |  | CP.3.2.8.3 |
|  |  |  | CP.3.2.8.4 |
|  |  |  | CP.3.2.8.5 |
|  | **CP.3.2.9**  **Training of referrers facilitates mobilisation** | Structures that promote normalisation | CP.3.2.9.1 |
|  |  |  | CP.3.2.9.2 |
|  | **CP.3.2.10**  **Challenges to be addressed in training** | Structures that promote normalisation | CP.3.2.10.1 |
|  |  |  | CP.3.2.10.2 |
|  |  |  | CP.3.2.10.3 |

### Table: Concise summary statements for Theme – Cognitive Participation - sub-node Enrolling and Supporting Delivery

[This sub-theme was not explored in the paper].

| **Tree nodes** | **Summary statement** |  | **Code** |
| --- | --- | --- | --- |
| **CP.3.3 Cognitive Participation/enrolling and supporting delivery** | **CP.3.3.1**  **Structured training** | Recognition of importance of well-structured training to enrol the coaches into the provider’s systems and programme content | CP.3.3.1.1 |
|  |  | Training sessions, supervision and weekly calls helped staff to come fully on board with the programme | CP.3.3.1.2 |
|  |  | Training that was regular and supported by management and peers assisted in keeping staff on board with the programme | CP.3.3.1.3 |
|  | **CP.3.3.2**  **Peer support** | Peer buddy for new coaches | CP.3.3.2.1 |
|  |  | WhatsApp group offered quick support to frontline staff | CP.3.3.2.2 |
|  | **CP.3.3.3**  **Structured communication channels** | Strong structure of internal communication which helped the staff to stay informed and feel supported | CP.3.3.3.1 |
|  | **CP.3.3.4**  **Contract liaison officer role** | Contract liaison officers liaise with GP practices to enrol them into referring patients onto the programme | CP.3.3.4.1 |
|  |  | Contract liaison officers support coaches with programme delivery | CP.3.3.4.2 |
|  | **CP.3.3.5**  **Teamwork** | Good teamwork was paramount for smooth programme delivery | CP.3.3.5.1 |
|  |  |  |  |

### Linking NPT construct COGNITIVE PARTICIPATION_ENROLLING & DELIVERY with descriptive and analytical themes

*To be read in conjunction with the summary statement for COGNITIVE PARTICIPATION – ENROLLING & DELIVERY*

| **NPT construct** | **Descriptive theme** | **Analytical theme** | **Code** |
| --- | --- | --- | --- |
| **CP.3.3 Cognitive Participation/enrolling and supporting delivery** | **CP.3.3.1**  **Structured training** | Structures that promote programme outcomes | CP.3.3.1.1 |
|  |  |  | CP.3.3.1.2 |
|  |  |  | CP.3.3.1.3 |
|  | **CP.3.3.2**  **Peer support** | Structures that promote programme outcomes | CP.3.3.2.1 |
|  |  |  | CP.3.3.2.2 |
|  | **CP.3.3.3**  **Structured communication channels** | Structures that promote programme outcomes | CP.3.3.3.1 |
|  | **CP.3.3.4**  **Contract liaison officer role** | Structures that promote programme outcomes | CP.3.3.4.1 |
|  |  |  | CP.3.3.4.2 |
|  | **CP.3.3.5**  **Teamwork** | Structures that promote programme outcomes | CP.3.3.5.1 |
|  |  |  |  |

### Table: Concise summary statements for Theme – Cognitive Participation - sub-node Mobilisation

[This sub-theme was not explored in the paper].

| **Tree nodes** | **Summary statements** |  | **Code** |
| --- | --- | --- | --- |
| **CP.3.5 Cognitive Participation/ Mobilisation** | **CP.3.5.1**  **Planning and preparation** | Plan for mobilisation process | CP.3.5.1.1 |
|  |  | Providers needed to prepare to receive the referrals | CP.3.5.1.2 |
|  |  | Changing from face-to-face to online delivery | CP.3.5.1.3 |
|  |  | Reasons for delay in mobilisation | CP.3.5.1.4 |
|  |  | Mobilisation was achieved while acknowledging some setbacks | CP.3.5.1.5 |
|  | **CP.3.5.2**  **Variation across areas** | Administration of CCG areas varied which impacted upon mobilisation | CP.3.5.2.1 |
|  |  | Pattern of roll-out differed across areas | CP.3.5.2.2 |
|  |  | Buy-in to the LCD pilot was reported as high in some places | CP.3.5.2.3 |
|  |  | Variation between areas as to how the referrer training was carried out | CP.3.5.2.4 |
|  | **CP.3.5.3**  **Differentiation from other programmes** | Increased workload associated with LCD programme | CP.3.5.3.1 |
|  |  | How the LCD programme differed from other programmes targeting this population was not always clear | CP.3.5.3.2 |
|  |  | LCD was differentiated from other programmes in some respects | CP.3.5.3.3 |

### Linking NPT construct COGNITIVE PARTICIPATION with descriptive and analytical themes for MOBILISATION

*To be read in conjunction with the summary statement for COGNITIVE PARTICIPATION*

| **NPT construct** | **Descriptive theme** | **Analytical theme** | **Code** |
| --- | --- | --- | --- |
| **CP.3.5 Cognitive Participation/ Mobilisation** | **CP.3.5.1**  **Planning and preparation** | Structures that promote programme outcomes  Structures that are barriers to programme outcomes | CP.3.5.1.1 |
|  |  |  | CP.3.5.1.2 |
|  |  |  | CP.3.5.1.3 |
|  |  |  | CP.3.5.1.4 |
|  |  |  | CP.3.5.1.5 |
|  | **CP.3.5.2**  **Variation across areas** | Structures that promote programme outcomes  Structures that are barriers to programme outcomes | CP.3.5.2.1 |
|  |  |  | CP.3.5.2.2 |
|  |  |  | CP.3.5.2.3 |
|  |  |  | CP.3.5.2.4 |
|  | **CP.3.5.3**  **Differentiation from other programmes** | Structures that promote programme outcomes  Structures that are barriers to programme outcomes | CP.3.5.3.1 |
|  |  |  | CP.3.5.3.2 |
|  |  |  | CP.3.5.3.3 |

### Table: Concise summary statements for Theme – Cognitive Participation - sub-node Gaps in training

[This sub-theme was not explored in the paper].

| **Tree nodes** | **Summary statement** | **Code** |
| --- | --- | --- |
| **CP.3.4 Cognitive Participation/gaps in training** | Participants did not identify many gaps in the training. | CP.3.4.1 |
|  | Felt ‘unsure’ about to deal with the unexpectedly high level of emotional eating in response to stress/depression among SUs. | CP.3.4.2 |
|  | Coaches were able to ask for support when they felt unsure. | CP.3.4.3 |
|  | Coaches said that they would go and research areas of uncertainty for themselves. | CP.3.4.4 |
|  | One participant reflected on doing things for the first time and how they felt unprepared. | CP.3.4.5 |
|  | Coach found it took a while to become proficient in real-life situations. | CP.3.4.6 |

### Linking NPT construct COGNITIVE PARTICIPATION with descriptive and analytical themes for GAPS IN TRAINING

*To be read in conjunction with the summary statement for COGNITIVE PARTICIPATION*

| **NPT construct** | **Descriptive theme** | **Analytical theme** | **Code** |
| --- | --- | --- | --- |
| **CP.3.4 Cognitive Participation** | **Gaps in training** | Structures that promote programme outcomes  Structures that are barriers to programme outcomes | CP.3.4.1 |
|  |  |  | CP.3.4.2 |
|  |  |  | CP.3.4.3 |
|  |  |  | CP.3.4.4 |
|  |  |  | CP.3.4.5 |
|  |  |  | CP.3.4.6 |
|  |  |  |  |
|  |  |  |  |
|  |  |  |  |

## Collective Action

### **Table: Concise summary statements for Theme – Collective Action**

| **Tree nodes** | **Summary statement** | **Code** |
| --- | --- | --- |
| **CA.1.1 Collective Action/Barriers to collective action/Service User** | The main barriers appear to disproportionately impact people from ethnic minorities (Asian or African Caribbean backgrounds), these multiple barriers relate to language and need for translation, also lack of family support and living in large families. | CA.1.1.1 |
|  | Lack of family support is a barrier which more commonly affects women from ethnic minorities. It is challenging for these women to manage the programme and still cook for their families. | CA.1.1.2 |
|  | Cultural barriers include Ramadan and Christmas which can be barriers to people doing the TDR phase. | CA.1.1.3 |
|  | Time commitments are a potential barrier for some SUs. | CA.1.1.4 |
|  | Veganism is a definite barrier to the programme because the TDR products are not suitable. | CA.1.1.5 |
|  | Taste preferences are a barrier for some SUs using the TDR products. | CA.1.1.6 |
|  | If TDR was not free of charge this would be a key barrier. | CA.1.1.7 |
|  | Trust as a potential barrier; it is important to build trust with the SU to increase engagement with the programme*.* | CA.1.1.8 |
|  | Barriers highlighted are mainly during the TDR phase of the programme. | CA.1.1.9 |
|  | Some providers seem more flexible and accommodating of potential cultural barriers than other providers. | CA.1.1.10 |
|  | Use of technology can be a barrier for older SUs. | CA.1.1.11 |
| **CA.1.2 Collective Action/Barriers to collective action/****Programme delivery (online)** | Some resources for SUs did not work well delivered online. | CA.1.2.1 |
|  | Using TDR for 12 weeks is difficult for SUs. | CA.1.2.2 |
|  | Coaches have to adapt activities and resources for online delivery. | CA.1.2.3 |
|  | Coaches have to adapt activities and resources for different sizes of online group format and also for online breakout rooms. | CA.1.2.4 |
|  | Coaches struggle with translating resources into other languages. | CA.1.2.5 |
|  | Issues with ordering TDR products and out-of-stock products (TDR) and issues with changing suppliers of these products. | CA.1.2.6 |
|  | Lack of guidance around the content of the programme for providers. | CA.1.2.7 |
|  | Stakeholders impact on programme delivery and it is important to have the ‘best’ people round the table, for each programme. |  |
| **CA.1.3 Collective Action/Barriers to collective action/Referring** | Lack of volume of referrals has been a barrier, with providers finding it challenging to fill the spaces they have on their programmes. | CA.1.3.1 |
|  | Within primary care there is a lack of understanding and training about how to make the referrals. | CA.1.3.2 |
|  | Medication adjustments and developing an agreed version of the referral form were biggest obstacles to implementation. | CA.1.3.3 |
|  | Providers need the ongoing support of the referrers throughout the programme. | CA.1.3.4 |
|  | Primary care has other priorities than making referrals to the programme. | CA.1.3.5 |
|  | Primary care capacity is the biggest barrier to referral and support of the programme. | CA.1.3.6 |
|  | Some patients have experienced difficulties getting referred onto the programme by their GP practices. | CA.1.3.7 |
|  | The cap on the number of referrals per practice was a hindrance to receiving referrals. | CA.1.3.8 |
|  | The referral process was negatively impacted by issues related to communication and engagement. | CA.1.3.9 |
|  | The lack of option to self-refer means the SU and provider rely on primary care involvement for referral. | CA.1.3.10 |
|  | Referral for LCD is more work for primary care due to medication adjustment. | CA.1.3.11 |
| **CA.1.4 Collective Action/Barriers to collective action/** **Perception of reasons for dropout** | There are many varied reasons proposed by providers for why SUs drop out of the programme.  Reasons stated include psychological reasons, chaotic circumstances, multiple life events, busy professionals, full-time workers, people with more social engagements, more work commitment that revolve around food, people with larger families, people who don’t work that have a lot of thinking time, being severely depressed, health issues, a lot going on at home, mental traumas, mindset. | CA.1.4.1 |
|  | Provider perception of reasons for drop out are all SU-related reasons. | CA.1.4.2 |
|  | Dropout can happen when the free TDR product phase is stopped at 12 weeks. | CA.1.4.3 |
|  | Dropout is not common and is usually due to serious health events and usually occurs before they have started the programme or in the initial phase. | CA.1.4.4 |
|  | Retention is better when GP only refer those most likely to stay in the programme. | CA.1.4.5 |
| **CA.2.1 Collective Action/Facilitators to collective action/Bridging role** | Providers support primary care with the referral process. | CA.2.1.1 |
|  | Achieving referrals requires engagement with various primary care stakeholders (pharmacists, practice nurses, individuals) which can be proactive or reactive. | CA.2.1.2 |
|  | Providers rely mainly on communication with non-clinical staff in GP practices. | CA.2.1.3 |
|  | Good communication including feedback and sharing of information, between contract liaison officers and GP practices, is vital to making the process of the programme work. | CA.2.1.4 |
|  | Contract liaison officers need to meet regularly and work closely with the site leads and the CCG to ensure change happens. | CA.2.1.5 |
|  | Delivery of the programme reduces pressure on GP practices as providers take more clinical responsibility for the patients. | CA.2.1.6 |
|  | More contact between providers and GP practices can lead to increased referrals. | CA.2.1.7 |
|  | A good relationship between provider and GP practices, through contract liaison officers, is important to ensure the patient referrals are appropriate. | CA.2.1.8 |
|  | Close and supportive teamwork: clinicians can access immediate frontline support from a colleague using online chats during live coaching sessions with service users. | CA.2.1.9 |
| **CA.2.2 Collective Action/Facilitators to collective action/Programme delivery (online)/Service user resources** | SU engagement with the pre-information resources is invaluable to the first appointment. | CA.2.2.1 |
|  | Providers try to replicate face-to-face delivery through remote delivery, using resources. | CA.2.2.2 |
|  | Including visual, active and tangible resources are important. | CA.2.2.3 |
|  | It is important to tailor the resources to individual service users. | CA.2.2.4 |
|  | It is important for providers to keep up to date with changing resources and where to locate them. | CA.2.2.5 |
| **CA.2.3** **Collective Action/Facilitators to collective action/** **Programme delivery (online)/Systems** | Delivery of the programme works well when all staff fulfil their role in the process of delivering the programme. | CA.2.3.1 |
|  | The operational system is critical to the success of the programme because the programme is complex, and it enables capture of data about the service users’ journey throughout the programme, both at critical timepoints and for different outcomes. | CA.2.3.2 |
|  | Independence from NHSE to work out programme design helps providers to develop the programme to suit them. | CA.2.3.3 |
|  | Remote delivery enables providers to support *more* service users. | CA.2.3.4 |
|  | Product delivery direct to service users’ homes is better for the providers. | CA.2.3.5 |
|  | More background information (in addition to clinical information) about each individual service user would help the coaching process. | CA.2.3.6 |
|  | Regular monthly team meetings and one-to-one meetings between provider and coach provides support for coaches to deliver the programme. | CA.2.3.7 |
|  | Coaches rely upon and support one another to deliver the programme. | CA.2.3.8 |
|  | Training of team members about the patient pathway is important to provide awareness of the ‘bigger picture’. | CA.2.3.9 |
| **CA.2.4** **Collective Action/Facilitators to collective action/ Programme delivery (online)/App** | The App enables regular and timely information between clinician and provider, about service users. | CA.2.4.1 |
|  | The App enables regular and timely feedback and support between provider and service user. | CA.2.4.2 |
|  | Feedback from service users is positive about using the App resources. | CA.2.4.3 |
| **CA.2.5 Collective Action/Facilitators to collective action/Referrals** | One-to-one programme delivery enables the provider to start delivery straight away (whereas group delivery model is reliant on waiting for a sufficient number of referrals). | CA.2.5.1 |
| **C.A2.6 Collective Action/Facilitators to collective action/Service user** | A working relationship between provider and service user, which includes building trust, taking time, positive encouragement, constructive feedback, developing confidence, is key to successfully delivering the programme. | CA.2.6.1 |
|  | Having a range of TDR products is important for the service user experience of the programme. | CA.2.6.2 |
| **CA.2.7 Collective Action/Facilitators to collective action/Programme content** | Revision of previous session topics help providers to check that service users have understood the programme content. | CA.2.7.1 |
| **CA.3.1 Collective Action/ Management of referrals and bookings/Service users** | At the point of booking, it is important to assess each service user’s commitment to and interest in the programme. (In addition to eligibility criteria, tolerance to the products and medication changes). | CA.3.1.1 |
|  | More written information on the risks of taking TDR and the eligibility criteria for TDR would benefit the management of referrals. | CA.3.1.2 |
| **CA.3.2** **Collective Action/ Management of referrals and bookings/Locality** | Remote delivery has enabled some providers, in conjunction with the relevant steering group, to open up referrals from GP practices out with the locality. | CA.3.2.1 |
|  | Referrals from secondary care are being developed. | CA.3.2.2 |
| **CA.3.3 Collective Action/ Management of referrals and bookings/GP Practice** | Providers rely on GP practice clinical systems for information on patients for referral. | CA.3.3.1 |
|  | GP practices response to information from providers about patients varies and impacts on the programme. | CA.3.3.2 |
|  | Problems with incomplete referrals are flagged to provider service managers who then get involved with the GP Practices. | CA.3.3.3 |

### Linking NPT construct COLLECTIVE ACTION with descriptive and analytical themes

*To be read in conjunction with the summary statement for COLLECTIVE ACTION*

| **NPT construct** | **Descriptive theme** | **Analytical theme** | **Code** |
| --- | --- | --- | --- |
| **CA.1.1 Collective Action** | **Barriers to collective action/Service User** | Personal characteristics and circumstances of SUs that are barriers to programme outcomes | CA.1.1.1 |
|  |  |  | CA.1.1.2 |
|  |  |  | CA.1.1.3 |
|  |  |  | CA.1.1.4 |
|  |  |  | CA.1.1.5 |
|  |  |  | CA.1.1.6 |
|  |  |  | CA.1.1.7 |
|  |  |  | CA.1.1.8 |
|  |  |  | CA.1.1.9 |
|  |  |  | CA.1.1.10 |
|  |  |  | CA.1.1.11 |
| **CA.1.2 Collective Action** | **Barriers to collective action/Programme delivery (online)** | Personal characteristics and circumstances of SUs that are barriers to programme outcomes | CA.1.2.1 |
|  |  |  | CA.1.2.2 |
|  |  |  | CA.1.2.3 |
|  |  |  | CA.1.2.4 |
|  |  |  | CA.1.2.5 |
|  |  |  | CA.1.2.6 |
|  |  |  | CA.1.2.7 |
| **CA.1.3 Collective Action** | **Barriers to collective action/Referring** | Structures that are barriers to programme outcomes | CA.1.3.1 |
|  |  |  | CA.1.3.2 |
|  |  |  | CA.1.3.3 |
|  |  |  | CA.1.3.4 |
|  |  |  | CA.1.3.5 |
|  |  |  | CA.1.3.6 |
|  |  |  | CA.1.3.7 |
|  |  |  | CA.1.3.8 |
|  |  |  | CA.1.3.9 |
|  |  |  | CA.1.3.10 |
|  |  |  | CA.1.3.11 |
| **CA.1.4 Collective Action** | **Barriers to collective action/** **Perception of reasons for dropout** | Personal characteristics and circumstances of SUs that are barriers to programme outcomes | CA.1.4.1 |
|  |  |  | CA.1.4.2 |
|  |  |  | CA.1.4.3 |
|  |  |  | CA.1.4.4 |
|  |  |  | CA.1.4.5 |
| **CA.2.1 Collective Action** | **Facilitators to collective action/Bridging role** | Structures that promote programme outcomes | CA.2.1.1 |
|  |  |  | CA.2.1.2 |
|  |  |  | CA.2.1.3 |
|  |  |  | CA.2.1.4 |
|  |  |  | CA.2.1.5 |
|  |  |  | CA.2.1.6 |
|  |  |  | CA.2.1.7 |
|  |  |  | CA.2.1.8 |
|  |  |  | CA.2.1.9 |
| **CA.2.2 Collective Action** | **Facilitators to collective action/Programme delivery (online)/Service user resources** | Systems that promote programme outcomes | CA.2.2.1 |
|  |  |  | CA.2.2.2 |
|  |  |  | CA.2.2.3 |
|  |  |  | CA.2.2.4 |
|  |  |  | CA.2.2.5 |
| **CA.2.3** **Collective Action** | **Facilitators to collective action/** **Programme delivery (online)/Systems** | Systems that promote programme outcomes | CA.2.3.1 |
|  |  |  | CA.2.3.2 |
|  |  |  | CA.2.3.3 |
|  |  |  | CA.2.3.4 |
|  |  |  | CA.2.3.5 |
|  |  |  | CA.2.3.6 |
|  |  |  | CA.2.3.7 |
|  |  |  | CA.2.3.8 |
|  |  |  | CA.2.3.9 |
| **CA.2.4** **Collective Action** | **Facilitators to collective action/ Programme delivery (online)/App** | Systems that promote programme outcomes | CA.2.4.1 |
|  |  |  | CA.2.4.2 |
|  |  |  | CA.2.4.3 |
| **CA.2.5 Collective Action** | **Facilitators to collective action/Referrals** | Systems that promote programme outcomes | CA.2.5.1 |
| **CA.2.6 Collective Action** | **Facilitators to collective action/Service user** | Personal characteristics and circumstances of SUs that promote programme outcomes | CA.2.6.1 |
|  |  |  | CA.2.6.2 |
| **CA.2.7 Collective Action** | **Facilitators to collective action/Programme content** | Systems that promote programme outcomes | CA.2.7.1 |
| **CA.3.1 Collective Action** | **Management of referrals and bookings/Service users** | Systems that promote programme outcomes | CA.3.1.1 |
|  |  |  | CA.3.1.2 |
| **CA.3.2** **Collective Action** | **Management of referrals and bookings/Locality** | Systems that promote programme outcomes | CA.3.2.1 |
|  |  |  | CA.3.2.2 |
| **CA.3.3 Collective Action** | **Management of referrals and bookings/GP Practice** | Systems that are barriers to programme outcomes | CA.3.3.1 |
|  |  |  | CA.3.3.2 |
|  |  |  | CA.3.3.3 |

## Reflexive Monitoring

### **Table: Concise summary statements for Theme – Reflexive Monitoring**

| **Tree nodes** | **Summary statement** | **Code** |
| --- | --- | --- |
| **RM.1. Reflexive Monitoring/ Internal feedback/ Ways to improve** | Prioritise more regular feedback and updates | RM.1.1.1 |
|  | Offer individual catch-up sessions | RM.1.2 |
|  | Importance of communicating progress across the programme | RM.1.3 |
| **RM.1.2 Reflexive Monitoring/ Internal feedback/ Wider System** | Initial referral rates low reportedly due to time required to build confidence of GPs in the programme | RM.2.1 |
|  | Referral processes created barriers to enrolment | RM.1.2.2 |
|  | Sometimes eligibility criteria were unclear, misinterpreted and seen to be unnecessarily limiting | RM.1.2.3 |
|  | Needed to develop new ways to deal with medical issues associated with the programme | RM.1.2.4 |
|  | Database lacks storage for qualitative data | RM.1.2.5 |
|  | A diverse range of delivery methods is key to making the programme accessible and sustainable | RM.1.2.6 |
|  | Choice of four TDR products reported as being inadequate for SUs | RM.1.2.7 |
|  | Concerns over future funding of the programme | RM.1.2.8 |
| **RM.2: Reflexive Monitoring/ Learning from Implementation process** | LCD was more challenging and complex to deliver than other interventions, creating some difficulties in practice | RM.2.1 |
|  | Compared to other similar programmes LCD required a higher level of recording and contact with SUs by the provider and more onus for recording fell to the SUs as well | RM.2.2 |
|  | Learning from phase 1 was reported to have been taken on board in the implementation of the new contracts | RM.2.3 |
|  | Data from the programme was available to adapt and shape it | RM.2.4 |
|  | Data from the programme was used to support SUs | RM.2.5 |
| **RM.3: Reflexive Monitoring/ Motivation of individual** | Motivation levels vary between individuals and within themselves and across the phases of the programme | RM.3.1 |
|  | Factors which supported SU motivation were reported to include: fitting the eligibility criteria, being in the right frame of mind, the phase of the programme, willing to make changes in their lifestyle, feeling/seeing the results quickly and mode of delivery | RM.3.2 |
|  | Factors which discouraged motivation were reported to include: the phase of the programme, low eligibility, disengagement, unmet expectations, live alone/without family support and group mode | RM.3.3 |
| **RM.4: Reflexive Monitoring/ Programme becomes part of normal practice** | Embedding within the provider, referrer and the wider context | RM.4.1 |
|  | Good communication systems that include informal and formal sessions with coaches and management | RM.4.2 |
|  | Programme success equated with further funding | RM.4.3 |
|  | Good training seen as increasing sustainability for SUs | RM.4.4 |
|  | Commissioning model is key to embedding the programme | RM.4.5 |
|  | Links with primary care are pivotal in embedding the programme | RM.4.6 |
|  | Links and contracts with other programmes strengthens the position of the provider with the wider stakeholders | RM.4.7 |
| **RM.5: Reflexive Monitoring/ Programme seen as a good thing** | Person centred approach taken | RM.5.1 |
|  | 12-month programme offers opportunity to enhance outcomes, including behaviour change, compared to shorter programmes | RM.5.2 |
|  | Common positive outcomes include: improved mood, weight loss, better diabetes management/remission, improved physical activity levels, increased awareness/knowledge of nutrition | RM.5.3 |
|  | Data collection has been thorough and provides clear evidence of positive outcomes | RM.5.4 |
| **RM.6: Reflexive Monitoring/ Reviewing LCD Programme** | Regular communication was paramount including update meetings for coaches; coaches individual meetings with senior staff; provider meetings with wider partners. | RM.6.1 |
|  | Good support structure for coaches in place including sharing feedback | RM.6.2 |
|  | Clear, user-friendly SU complaints procedures in place | RM.6.3 |
|  | Good recording structures | RM.6.4 |
|  | Data available to adapt services and processes | RM.6.5 |
|  | Shared information and learning from other programmes targeting diabetes clarifies the fit of LCD into wider provision | RM.6.6 |
| **RM.7: Reflexive Monitoring/ Ways to improve equity of programme** | Adapt materials/examples to cultures represented within the group | RM.7.1 |
|  | Work with Black and Minority Ethnic SUs was patchy but with little recognition that more work needs to be done | RM.7.2 |
|  | More flexibility in delivery methods – not just face to face/group/digital but a mix; virtual delivery was seen to complement in-person delivery | RM.7.3 |
| **RM.8: Reflexive Monitoring/ Ways to improve products** | Allow testing of products by SUs before choosing so that returns/exchanges are reduced | RM.8.1 |
|  | Provide SUs with a wide number of products | RM.8.2 |

### Linking NPT construct REFLEXIVE MONITORING with descriptive and analytical themes

*To be read in conjunction with the summary statement for REFLEXIVE MONITORING*

| **NPT construct** | **Descriptive theme** | **Analytical theme** | **Code** |
| --- | --- | --- | --- |
| **RM.1. Reflexive Monitoring** | **Internal feedback/ Ways to improve** | Structures that promote normalisation | RM.1.1.1 |
|  |  |  | RM.1.2 |
|  |  |  | RM.1.3 |
| **RM.1.2 Reflexive Monitoring** | **Internal feedback/ Wider System** | Structures that are barriers to normalisation | RM.2.1 |
|  |  |  | RM.1.2.2 |
|  |  |  | RM.1.2.3 |
|  |  |  | RM.1.2.4 |
|  |  |  | RM.1.2.5 |
|  |  |  | RM.1.2.6 |
|  |  |  | RM.1.2.7 |
|  |  |  | RM.1.2.8 |
| **RM.2: Reflexive Monitoring** | **Learning from Implementation process** | Structures that promote normalisation  Structures that are barriers to normalisation | RM.2.1 |
|  |  |  | RM.2.2 |
|  |  |  | RM.2.3 |
|  |  |  | RM.2.4 |
|  |  |  | RM.2.5 |
| **RM.3: Reflexive Monitoring** | **Motivation of individual** | Factors that promote normalisation  Factors that are barriers to normalisation | RM.3.1 |
|  |  |  | RM.3.2 |
|  |  |  | RM.3.3 |
| **RM.4: Reflexive Monitoring** | **Programme becomes part of normal practice** | Structures that promote normalisation | RM.4.1 |
|  |  |  | RM.4.2 |
|  |  |  | RM.4.3 |
|  |  |  | RM.4.4 |
|  |  |  | RM.4.5 |
|  |  |  | RM.4.6 |
|  |  |  | RM.4.7 |
| **RM.5: Reflexive Monitoring** | **Programme seen as a good thing** | Factors that promote normalisation | RM.5.1 |
|  |  |  | RM.5.2 |
|  |  |  | RM.5.3 |
|  |  |  | RM.5.4 |
| **RM.6: Reflexive Monitoring** | **Reviewing LCD Programme** | Factors that promote normalisation | RM.6.1 |
|  |  |  | RM.6.2 |
|  |  |  | RM.6.3 |
|  |  |  | RM.6.4 |
|  |  |  | RM.6.5 |
|  |  |  | RM.6.6 |
| **RM.7: Reflexive Monitoring** | **Ways to improve equity of programme** | Structures that promote equity | RM.7.1 |
|  |  |  | RM.7.2 |
|  |  |  | RM.7.3 |
| **RM.8: Reflexive Monitoring** | **Ways to improve products** | Factors that promote normalisation | RM.8.1 |
|  |  |  | RM.8.2 |

## Equity

### **Table: Concise summary statements for Theme – Equity**

| **Tree nodes** | **Summary statement** | **Code** |
| --- | --- | --- |
| **E.1. Equity/cultural competence of the programme** | When talking about cultural competence it is mainly in relation to providing different food choices and changing the programme to fit around festivals such as Ramadan. | E.1.1 |
|  | There is relatively less discussion about wider cultural issues (than providing culturally appropriate food options and recipes) including how people from different cultural backgrounds view health. | E.1.2 |
|  | TDR might be more of a challenge to people from minority ethnic groups where traditional food is a big part of their culture, where women do all the cooking in the home and for women living in larger families. | E.1.3 |
|  | Having service managers, nutritionists, dietitians, and health coaches from ethnically diverse backgrounds can provide insight within the service and support to minority ethnic group participants. | E.1.4 |
|  | Cultural competence seems to be viewed as something to be considered if, and when, needed. | E.1.5 |
|  | The need for a programme to be culturally competent seems to vary across different providers and this is related to ethnic diversity of geographic areas and perhaps due to lower referrals of people from minority ethnic groups. | E.1.6 |
|  | Providers will have to work with GP practices to do targeted referral to do a ‘language group’. | E.1.7 |
|  | Individual coaches can make the programme more culturally competent by personalising it, for example by swapping Western recipes for more culturally appropriate recipes. | E.1.8 |
|  | Language is seen as the main cultural barrier. See E.6 | E.1.9 |
|  | Group support from other service users can help with cultural differences. | E.1.10 |
| **E.2. Equity/differences between areas** | There is a difference in what is offered between areas and so it depends on where a person lives as to what they receive (group, digital or one-to-one). | E.2.1 |
|  | Some areas have no offer at all for people with type 2 diabetes. | E.2.2 |
|  | The areas are very different in terms of population demographics. Face-to-face models have geographic boundaries on who they invite to the programme, and so this means, in some areas, there is limited diversity in socioeconomic status or ethnicity. | E.2.3 |
|  | The areas are very different in terms of size, which seems to vary according to the number of contracts held by each provider. | E.2.4 |
|  | Offering different programme models, with a choice of evening and weekend programmes, reduces time restriction barriers and captures the working population, which potentially reduce socioeconomic inequity. |  |
|  | Providers do look at areas where they are not getting many referrals, to see if they need to focus more in these areas. | E.2.5 |
|  | In areas where there is ethnic diversity this is not fully reflected in the SU group. | E.2.6 |
| **E.3. Equity/digital engagement** | Online groups increase attendance compared to face-to-face. | E.3.1 |
|  | Both coaches and service users like the remote approach (according to providers). | E.3.2 |
|  | Coaches can see people more quickly and easily online. | E.3.3 |
|  | Contract liaison officers had a very different view of being online and felt that they cannot give as much support or as much engagement via Teams compared to face-to-face. | E.3.4 |
|  | Online delivery helps with engagement because it is easier for some service users to discuss certain issues in depth when in online groups, such as mental health and comfort eating, compared to being face-to-face in a group setting. | E.3.5 |
|  | The chat function in Teams provides a useful additional option for service users who do not want to talk and this aids engagement. | E.3.6 |
|  | Remote delivery appeals to people who wouldn’t usually accept care because they can do it from home and have family support with them. | E.3.7 |
|  | Digital engagement is very useful for clinical practitioners as they get more frequent and accurate information compared to NHS appointments every 2-3 months that also have the issue of recall. | E.3.8 |
|  | Online delivery is useful to complement in-person or as part of a suite of delivery options but is not suitable for everyone. | E.3.9 |
|  | Digital engagement is an issue for people living in more remote areas. | E.3.10 |
|  | Digital delivery has increased engagement from working age men as they don’t need to take time off work to attend. | E.3.11 |
|  | The cap of 65 years for programme entry means that most service users are of working age and less impacted by digital exclusion. | E.3.12 |
|  | Positive statements about digital delivery are about inclusion and also about mobilisation. | E.3.13 |
|  | Providers seem to assume being ‘tech savvy’ means being able to use the App. | E.3.14 |
|  | Assumes digital delivery is accessible to all as everyone can get online. There is an option to do the programme modules without a smart phone so service users can engage with remote delivery if they are familiar with using websites but do not want to use the App. | E.3.15 |
|  | Digital engagement varies greatly between service users, some send a lot of messages (App) and expect providers to be on hand all the time whereas providers have to nudge some users. | E.3.16 |
|  | Use of the App is not always a true reflection of engagement as service users can be engaged with the programme but not chatty using the App. | E.3.17 |
|  | Users that log and access the learn content (App) do better on the programme (unclear how ‘better’ is defined). | E.3.18 |
|  | The App provides a means for both the service user and provider to get and keep engaged through tracking and reminders and logging measurements. | E.3.19 |
|  | A challenge with the App is getting service users to upload photos – this really helps providers with portion size. | E.3.20 |
|  | Options with non-engagement with the App is conversion to phone calls or a hybrid approach whilst **trust** is built between service user and coach. | E.3.21 |
|  | It takes times to build up service users’ confidence in using the App but they get used to it and start to use it. | E.3.22 |
| **E.4. Equity/digital pathway** | Service users receive the same amount of care from a dietitian and coach regardless of pathway although it appears that frequency and durations of individual contacts may differ. | E.4.1 |
|  | Face-to-face (on the digital programme) and digital option only differ in that the face-to-face option is a Teams videocall and the digital option is a phone call. | E.4.2 |
|  | On the digital pathway the contacts are more for support and are user-led. | E.4.3 |
|  | The digital offer may differ between providers. | E.4.4 |
| **E.5. Equity/referral process** | Evidence from senior team members and contract liaison officers suggest there have been lower referrals from BAME groups. | E.5.1 |
|  | Equity of the referral process is impacted by how much practice staff ‘sell the programme’ to patients. | E.5.2 |
|  | Some areas at the beginning put a cap on referrals per practice to be equitable but this was not needed and was a hindrance to getting the referrals through in some practices. | E.5.3 |
|  |  | E.5.4 |
| **E.6. Equity/language** | Language is seen as the main cultural barrier. See E.1.1. | E.6.1 |
|  | Some service providers have Urdu and Hindi speaking group sessions. | E.6.2 |
|  | There is disparity across service providers in terms of what is offered to overcome any language barriers. | E.6.3 |
|  | In some areas there was difficulty in getting enough people to start an Urdu group. | E.6.4 |
|  | Usually a daughter or husband helps with any language barriers during telephone calls between Urdu speaking women and the service provider. | E.6.5 |
|  | One provider does not provide sessions in any other language other than English and so cannot accept non-English speaking people onto the programme. | E.6.6 |
|  | Depending on a family member or friend for translation is a barrier to accessing and engaging with the programme. | E.6.7 |
|  | Translating technical wording, both in the physiology sessions and the TDR product information) can be challenging for the coach even when the coach is fluent in the language of the service user. | E.6.8 |
|  | Equity related to language is not embedded into any of the programmes and is viewed as an ‘add-on’ to be considered when the need arises. | E.6.9 |
|  | Sometimes Teams can be an extra barrier if there is a time lag and there is also a slight language barrier, the coach can address this by making a phone call. See also E.3. | E.6.10 |
|  | The App has an option to switch resources into other languages, Hindi was reported. See also E.3. | E.6.11 |
|  | Service users where English is a second language, would struggle without extra help taking their measurements and relaying them to the coaches over the phone. | E.6.12 |
|  | Majority of service users where English is a second language, are not able to read English and struggle with the written resources. More visual resources could help. | E.6.13 |
|  | Service users where English is a second language, heavily rely on their ‘support person’ across various aspects of the programme (phone calls, taking measurements, understanding the workbooks, reading the TDR products, the personal action plan). | E.6.14 |
| **E.7. Equity/product** | Providers talk positively about TDR and fibre being funded and so patients do not have to pay for the product and that this means ‘equality of access’. | E.7.1 |
|  | Purchasing of the TDR product would be a barrier to joining the programme for service users. | E.7.2 |
|  | The programme would not be sustainable without government funding of the TDR product. | E.7.3 |
|  | Perhaps service users in more affluent areas could afford to pay for their TDR although this would be based on geography and not on individual circumstances. | E.7.4 |
|  | The TDR product is seen as very important to the success of the programme. | E.7.5 |
|  | There is no vegan option of the TDR product. | E.7.6 |
| **E.8. Equity/sample** | There is underrepresentation from people from Asian ethnicities especially considering the prevalence of diabetes in this population. | E.7.7 |
|  | The most underrepresented groups are ethnic minority groups, but this is also representative of the area some of the providers are working in. | E.7.8 |
|  | Providers say they are struggling to get referrals from minoritised ethnic groups, and it is not the case that there are lots of referrals from people from these groups that then do not start the programme. | E.7.9 |
|  | The programme has engaged an equal number of men to women which is unusual for weight management programmes. | E.7.10 |
|  | Data is examined using deprivation quintiles including engagement and participation at referral, individual assessments, starting a programme, and retention by IMD quintile. There is no outcome data yet by sociodemographic breakdown. | E.7.11 |
|  | Service provider models that were meant to be face-to-face are limited by proximity to the hospital site; this is a barrier to producing an ethnically diverse sample. | E.7.12 |
|  | There seems to be a range of representation differing across the providers (maybe related to geography) and across the models. | E.7.13 |
|  | Targeted work within practices would help to produce a more representative sample of referrals but practices do not have the time or capacity to work on targeted referrals. | E.7.14 |
|  | Targeted work with practices is necessary to meet the need for non-English language groups. | E.7.15 |
|  | The digital model might attract a younger sample because this age is more used to using technology and the digital model enables the service user to message at whatever time and get feedback quickly (the next day). | E.7.16 |
|  | The digital model does not have a culturally diverse sample. | E.7.17 |
|  | There is a fairly even spread of drop out across the programme except for lowest social quintile; so there is not an inverse gradient across all the quintiles. | E.7.18 |

## Person centredness

### **Table: Concise summary statements for Theme – Person centredness**

| **Tree nodes** | **Summary statement** | **Code** |
| --- | --- | --- |
| **F.1. Person centredness/Coach and service user relationships** | It is very important for all providers to establish a relationship with individual service users and understand their lives so that they can individualise their care and delivery by adapting the care plan to suit each service user. | F.1.1 |
|  | The programme is personalised in the practical application of the programme by individual coaches. Providers of one-to-one programme models or one-to-one options, like the ability to personalise the programme and tailor the programme sessions to individual service user needs. | F.1.2 |
|  | Providers of both the group and the digital offer, want one-to-one calls, to be able to get to know their service users and to understand what they need from them. | F.1.3 |
|  | Providers get a sense of satisfaction from regularly following-up with the same service users and supporting them through their 12-month journey. | F.1.4 |
|  | Providers acknowledge that behaviour change takes a long time, and they get a sense of satisfaction from seeing this behaviour change in individual service users that they have supported through their journey. | F.1.5 |
|  | Providers enjoy the opportunity the programme gives them to build a strong relationship with service users. | F.1.6 |
|  | Providers believe that the strong relationship they build with service users, contributes to their success on the programme. | F.1.7 |
|  | A good relationship between coach and service user can be developed due to regular, long-term, and consistent support. Having a coach for a year means that the coach can build a good relationship with the service user. Coaches can provide more attention and support than can be offered in the NHS. | F.1.8 |
|  | Service users share their experiences with the coach who supports the service user through general life events and shows them how to problem solve and keep on track with the programme. | F.1.9 |
|  | The App has changed the relationship that service users have with their coach because it enables direct feedback between coach and service user especially regarding clinical measurements. The coaches like receiving measurements direct form the service users, to review. Coaches can directly support via the App and keep service users engaged and self-tracking also keeps service users motivated. | F.1.10 |
|  | The App enables the coach to give individual feedback as they can see through the service user sending food photos, exactly what they are eating and the portion sizes. | F.1.11 |
|  | Relationships between coaches and service users are influenced by the type of programme model. | F.1.12 |
|  | There is a hybrid approach used initially with the App, using phone calls whilst coaches build trust between them and the service user. | F.1.13 |
| **F.2. Person centredness/Making the programme work for the individual** | Providers recognise that not every session on the programme will suit every service user, but each session will work for some. | F.2.1 |
|  | Service users use the programme sessions in different ways and for different reasons. Some service users just want to check-in and report their measurements and are not interested in the behaviour change, whereas other service users want to build rapport with their coaches. | F.2.2 |
|  | Coaches think that their service users prefer them to adapt the programme to real-life everyday issues rather than talking about theoretical behaviour change concepts. | F.2.3 |
|  | Providers of one-to-one sessions say they can adapt what sessions they do, how they carry them out and even how long the sessions are for each service user. This feels important to the coaches and to their perception of service user needs. | F.2.4 |
|  | Online sessions are beneficial to both service users and coaches because they provide flexibility. | F.2.5 |
|  | The biggest benefit of the digital model is that there are no time restrictions for the service user. | F.2.6 |
|  | Providers acknowledge that service users join the online sessions when they are also busy doing other things. | F.2.7 |
|  | Coaches say it is important to provide space at the end of the programme sessions for service users to ask and discuss anything they want to, and this is where service users contribute quite a lot; after the formal learning part of the session. | F.2.8 |
|  | Tailoring the programme to the needs of each service user is a key factor in making the programme work well. | F.2.9 |
|  | Tailoring occurs throughout the programme (but perhaps outside of the formal programme sessions), from initial assessment by the Patient Support Team, the Initial Interview form, and the Personalised Action Plan which considers their culture, lifestyle, budget, tastes and capabilities. | F.2.10 |
|  | Service users with co-morbidities receive more support from staff with regards to medical monitoring. | F.2.11 |
|  | It is not the content of the programme sessions but the delivery of them that makes them person-centred. Adapting the sessions to make them relevant to all service users is key. | F.2.12 |
|  | Providers provide the information and service users have the choice to reflect on how they can use that information and to engage and to make behaviour changes. | F.2.13 |
|  | Group sessions are especially valuable to service users who do not have a family/support network; for these people the longer gap between session during the maintenance phase is more of a challenge. | F.2.14 |
|  | The programme provides sustainable change for individual service users as it provides service users with the skills to continue leading healthier lifestyle after the programme ends. | F.2.15 |
| **F.3. Person centredness/peer support between service users** | Service users support each other during the group sessions, and this leads to successful outcomes for all service users. | F.3.1 |
|  | The group sessions are especially beneficial to service users when there is a range of service users from different backgrounds and ethnicities and where English is a second language for some, because service users support each other. | F.4.1 |
|  | Service users in the group sessions, support each other because they are in the same situation. This encourages service users to share their experiences within the group session. There is a sense of camaraderie between service users because they are on the same journey. | F.4.2 |
|  | The group chat message function is being used by service users to send positive messages to each other during the group sessions and this in turn, encourages verbal sharing of stories with the coach. | F.4.3 |
|  | The coach learns more about what works and what doesn’t work through this dialogue between service users during group sessions. | F.4.4 |
|  | There is discussion between service users at key timepoints during the group session, both in support for each other during the ‘progress review’ at the start of the session, but also in discussion arising during the coach’s presentation. | F.4.5 |
|  | Peer support is another level of support for service users, this is not viewed as an ‘add-on’ but as very important. | F.4.6 |
|  | Peer support is important in keeping service users motivated and engaged and perhaps prevents drop out. | F.4.7 |
|  | When service users do not attend group sessions they let other know on the WhatsApp group which is then shared during the group sessions, so coach’s learn information about non-attenders through the sharing of information from the WhatsApp chat. | F.4.8 |
|  | There is a ‘community forum’ on the App where services users are encouraged to share. | F.4.9 |
|  | Peer support is also developed outside of the group sessions, such as WhatsApp groups. | F.4.10 |
|  | Peer support outside of the group sessions is especially helpful to service users during the maintenance phase, when time between group sessions is longer. | F.4.11 |
|  | Not all service users join external online peer support groups. | F.4.12 |
| **F.5. Person centredness/Integrating programme, service user needs and healthcare systems** | There is more communication between the service user, provider doctor, clinical lead, medical director and GP centres when the service user has co-morbidities and requires medication monitoring. | F.5.1 |
| **F.6. Person centredness/Individual service user calls outside group session** | Individual calls between the coach and the service user are important for the service user to be able to discuss sensitive issues such as ‘mental health’ away from the group sessions, and in more depth, with the example of ‘comfort eating’. | F.6.1 |
|  | Individual calls between the coach and the service user are important for collecting measurements. | F.6.2 |
|  | Individual calls between the coach and the service user are important, to learn more about each individual service user and their needs, but these calls are viewed as a short catch-up; however, keeping it short is sometimes a challenge. | F.6.3 |
|  | Individual calls between the coach and the service user vary in frequency and intensity according to mode (telephone, app) and what stage the SU is at in the programme. | F.6.4 |

## Additional codes not reported in main text- Within the Cognitive Participation theme

#### Preparation for mobilisation

The provider met with staff in the localities and devised a plan for how to implement the intervention.

“So as soon as we get the go ahead from the NHS, we set up meetings with the individual locations, get a mobilisation plan put in place; so we have our mobilisation team and our mobilisation manager that helps us with that. Where we then set up regular meetings, either on a weekly or fortnightly basis initially, with the key contacts in the new contract areas, to talk about the physicality of mobilising that, that contract. (PFG10, PS45)

However, it was noted that there was an increased workload associated with mobilising and sustaining the LCD programme compared with similar programmes.

“… it's also actually more work, and so we didn't know how that was going to play out in practice, especially when everyone was busy.” (PFG09, PS41)

Due to variation in decisions by Integrated Care Boards, the pattern of roll-out differed across localities.

“So, we took a decision to change things quite significantly, beyond the move to virtual, and open it up. This was in conjunction with the steering group in [city 2], opening up more broadly to any practice in [city 2] could refer who was interested because it didn't matter where people were. You didn't have to have that geographical clustering with an in-person programme.” (PFG09, PS41)

However, in another focus group, participants also talked about a phased roll out.

“… each area was quite different in how they wanted to mobilise. You know, some of them did like a phased rollout approach.” (PFG03, PS15)

If providers were delivering the programme across CCG boundaries this created differences in the mobilisation process.

“… so it was a little bit different in their approach to perhaps some other areas where there was one CCG. They had to have some extra thinking and planning around how that would work. So yeah, it was a lot, initially it was about meeting with the sites quite regularly to discuss how that would work.” (PFG03, PS15)

It was recognised that there were advantages and disadvantages to both patterns of roll-out.

“That was helpful in some ways, but unhelpful in others because it might have removed the urgency and focus for practices to refer, but it opened it up to lots more who otherwise would not have been able to.” (PFG09, PS41)

Another aspect of variation was the level of buy-in to the programme. It was higher in some localities compared to others. This also impacted upon the mobilisation process.

A senior manager in one provider reported that local buy-in from stakeholders was unusually high, although the reasons for this were not spelt out.

“I probably had one of the best initial steering group meetings in terms of representation that I've ever seen in in, you know, dozens of mobilisations, and it was very impressive. It soon whittled down to a core group, but it was a, it was a very impressive set of stakeholders being engaged from the outset.” (PFG09, PS41)

The senior manager went on to state that this high level of buy-in created pressure and momentum to deliver the LCD programme.

“… that influenced our plans quite a lot because we didn't know, there was a lot of pressure on primary care when we were starting to roll this out, so that was a massive change …” (PFG09, PS41)

The aim of the referrer training was to inform them about the specific requirements of the LCD programme and how it differed from other programmes for this population. Again, there was variation between areas as to how the referrer training was carried out, whether it was one event that everyone was expected to attend or whether it was ongoing as required.

“… we had our initial training session for people, I had I think was it 120 people or something [PS39] wasn’t it, maybe 115 or something which was the most I've ever seen at a mobilisation training for any of these services, including the NDPP which runs at a much larger scale.” (PFG09, PS41)

“I think another important part of that mobilisation was the training for the referrers, so actually getting them to a point where they knew about the programme, they understand the deprescribing, they know kind of how to refer and that's been, I mean in [area 2] they’ve continuously done that referrer training, whereas in other areas they did, you know, one big one.” (PFG03, PS15)

Key aspects of preparing for mobilisation from the providers’ perspective were to be ready to receive referrals and to deliver the programme.

“So, when we initially had, you know, all the initial referrals, it was all brand new to everyone. We all pretty much, the diabetes practitioners and PST [Patient Support Team], got pretty much the same training and the same introduction to the programme. And then yeah, I think it was just on boarding all the patients.” (PFG06, PS24)

This was often accomplished by drawing in staff with experience of other programmes for this population. The difference between these programmes and the LCD was not always clear to frontline staff.

“… it's a bit of a funny one 'cause I was a diabetes practitioner on a different programme. So we have our [programme name] programme and I was a DP [diabetic practitioner] on that, so I think I was kind of asked to support; so when for example [PS28] and other people who were coming on as the, the first ever diabetes practitioners on LCD, I was asked to support with I guess my experience of working with diabetic service users.” (PFG06, PS16)

It was noted that the LCD programme differed in terms of the greater clinical and medication emphasis that required the provider to have the expertise of a medical director within the team.

“… a bit like some of the other providers, who weren't delivering more clinical services, we had to get a medical director on board, and recruit her in this case. And also flesh out what that role actually was and what it looked like in reality, as opposed to on paper, and how it all worked together. (PFG09, PS41)

There were a number of reasons for delay in mobilisation. This increase in medical oversight by the provider caused some delay for one provider, while processes were agreed, due to nervousness around giving some clinical responsibilities, that usually lay with GP practices, to the providers instead.

“… although generally, mobilisation was smooth, we had two significant glitches. One glitch was around the medication adjustment and the medicines management team in [city 2]. Again, this is a change from other mobilisations, who don't normally interact with medicines management, but because of the medications review, that was required and, and they had quite significant concerns which slowed down the mobilisation, and we had to set up calls with [colleague] at NHSE and other things, which was a bit of a nuisance, but he was very obliging. And eventually it turned out they made no changes whatsoever, which was a bit frustrating but one of those things. But it set things back by, probably delayed our launch by four weeks.” (PFG09, PS41)

Other reasons included the onset of the pandemic, which caused delay and uncertainty and disrupted mobilisation.

“… the first different thing was that mobilisation was meant to start and then got pushed back by, so a few months. I want to say about six but not, you know, give or take, and during that period there was a bit of uncertainty.” (PFG09, PS41)

In one area, there was also a miscommunication between the software engineers and the other stakeholders, whereby it was understood that the LCD software was ready to upload centrally but the reality was that each GP practice had to upload it themselves.

“… the [IT] systems [managers] who were very involved in the steering group, but when they said the search was available to all the GP practices, the clinical director, everyone else in the steering group and we assumed that meant it was easy for them to access, as opposed to it actually meant they had to upload it practice by practice, and it hadn't been done centrally after all.” (PFG09, PS41)

The result of this miscommunication between the software engineers and the practitioners was further delay, which was compounded by the pandemic.

“… we hadn't picked that up, because different versions of EMIS and Systm One [software used by GP practices] have different, and different areas have different ways that they interact centrally versus practice by practice, so it’s perfectly possible they [GP practices] hadn't done it. So that caused another three weeks of delay, which put us right into, into the wave two [of the pandemic], which was a pain.” (PFG09, PS41)

However, this time of delay and feeling of being becalmed preceded a brisk increase in referrals.

“So, it kind of took the wind out of the sails as soon as we just got going, and then it vroom.” (PFG09, PS41)

Due to the pandemic, there was a change from face-to-face to online delivery.

“There was some discussion with NHS England about what the adaptations for virtual delivery would be.” (PFG09, PS41)

This created the need to adapt the plan for delivery.

“I would say that the original idea was obviously for this to be a face-to-face model. So unfortunately, due to you know everything we've all experienced in the last 18 months, that's just not been possible.” (PFG04, PS11)

One coach (diabetic practitioner) spoke about how the provider supplied them with good technical support to make this switch.

“I think the good thing is that we do have technology, you know. I know it plays up sometimes, but having you know the company provided all of us with laptops as well, so we can then be on camera so the company did the best that they can by making this a face-to-face model …” (PFG04, PS11)

Nevertheless, the coach expressed some reservations about the lack of being face-to-face with the SU but suggested that, when using cameras online, it was a good substitute.

“… even though we're not with people. So, it's still a personal touch. It's not over the ‘phone and we can see people, you know most people have their cameras on, so we can see who they are.” (PFG04, PS11)

The coach acknowledged that there was likely to be some disappointment for SUs about the lack of face-to-face delivery, but that due to the pandemic it was unavoidable and still provided a suitable approach.

“… so it's probably not designed the way they wanted it to, because it's not face-to-face. But for the circumstance, in my opinion, it's as good as. You just can't, yeah, you're just not face-to-face and with someone in a room, if that makes sense, but you're still in a session.” (PFG04, PS11)

Mobilisation was achieved while there was acknowledgment that there had been some setbacks.

One provider explained how mobilisations normally work well in parts but also have setbacks and this was no different for the LCD programme.

“Extra challenges, extra opportunities … there were lots of things that worked really well, with a couple of glitches, but there are very few mobilisations that have everything that works well even when you think you're on top of it.” (PFG09, PS41)

#### Supporting enrolment and delivery

It became clear during analysis that middle managers functioned in a bridging role between the commissioner, senior management, multidisciplinary team (MDT) and frontline staff. Their main aims were reported as maintaining the contract, delivering the programme effectively and priority setting.

“They [Contract Liaison Officers (CLOs)] work together as a collective to decide what's the priorities … And you know these guys might say to me, look, I think we should do something else, and they'll come to me with suggestions.” (PFG05, PS21)

This also involved creating solutions when challenges arose.

“… what the contract liaison officers have to do is, although they’re working quite closely with the practices, obviously that's always linking back to the project leads within the area, the CCG [Clinical Commissioning Group], feeding that back to them because obviously we can, we can do a certain amount our side, but often need to work really closely with them to drive any of those kind of changes or efficiencies through.” (FG03, PS15)

“… it's understanding what are their [frontline staff] support needs … to deliver the best that they can to make the programme you know, work as effectively as it can do.” (PFG03, PS13)

Teamwork was identified by participants as another factor in supporting enrolment and delivery. Good teamwork was recognised as paramount for smooth programme delivery.

“Sometimes, when you got a, an unusual customer that you wanted some really good mentoring and advice, then I used to give [PS8] a quick call.” (PFG02, PS06)

“So, if the CLOs have done their part of setting up the group, almost listening to us as DPs [diabetic practitioners] of when we're available. And then you know the training, so in terms of the slides, how we deliver it, how we upload it to Microsoft Teams, and then once again the work that the lead DPs do, PST do, the patient support team. If all of that’s done right, then for me personally, in my opinion the delivery side is then very simple and very smooth...” (PFG04, PS11)

“… we as a group ironed out any problems pretty quickly and changed procedure if need be.” (PFG04, PS17)

Peer support was identified by participants as a way that staff engagement was facilitated. This included being allocated peer buddies for new members of staff and creating WhatsApp groups, which enabled fast discussion and support at times of need.

“[PS8] was my buddy and she really helped every time I got stuck. Which was probably about seven times every, every day, I used to give her a call. That was a joke.” (PFG02, PS06)

“Then it’s like am I equipped to deal with this? And it's all about you know, keeping, keeping yourself calm and if you're unsure about anything, either contacting other coaches, putting it on our sort of chat group, WhatsApp group, to ask for advice from other coaches, or just doing your own research so you can go back, you know a little bit more prepared the next time.” (PFG02, PS08)

“So, if they're live coaching a patient and they need that support, they can get it from a colleague there and then, which I think is a, is a really a big positive of [provider] and it shows how close and supportive our teams are of one another.” (PFG10, PS44)

### Within the Collective Action theme

Delivery of the programme reduced pressure on GP practices as providers took more clinical responsibility for the patients.

“I think it does make it a lot easier for them because we so like for example the patients do their own blood sugar readings and their own blood pressure readings, and they take their own weight measurements, so they don’t have to go into the practice for that. And we submit the health incidents as well so if they do have high blood sugar readings or blood pressure readings, unless they are severely high and they tend to get passed on to our multidisciplinary team. So yeah, a lot of the adverse effects and things like that we do take responsibility for to some extent. So yeah, I think it does relieve a lot of pressures.” (PFG06 PS28)

## Extended quotes offering more detail

### Within the Coherence theme

#### Facilitators to coherence:

Providers also spoke of the longer-term (52 weeks) support which differentiates the programme.

“I think that's been really key, is that because there is that kind of very frequent level of support over a year long period, it really enables them to embed those new habits into their lives.” (PFG10, PS49)

### Within the Cognitive Participation theme

#### Feedback loops

Several types of feedback were discussed in the focus groups:

- Internal - Routine data collection (quantitative)
- Internal - Performance of coaches (observational)
- External - From SUs/ other stakeholders to provider (qualitative)
- External – From provider to SUs/other stakeholders

Feedback was used by provider organisations for service improvement, maintaining standards, encouragement of staff and professional development. One provider conducted observations on their coaches to check if quality standards were being maintained.

“… to spot check the quality of those one to ones, we do observations on our coaches ... observations take place so that we can ensure the consistency and the quality approach of that delivery.” (PFG13, PS67)

From the coach’s perspective it was a good personal development structure.

“I was going to say it's [personal] development, but a conversation afterwards just to go through the feedback that you've got.” (PFG04, PS19)

This coach recognised how it helped them to deliver a better service to the SU.

“So it goes both ways, it's the support to SUs [service users], the business to be able to make sure the delivery is correct and on how it's done, and then any feedback on that, and to develop that, and then of course your own personal development in making sure that you're doing [it right], you know, this is why we're here enjoying our role to be able to support the SUs going forward.” (PFG04, PS19)

Routine data collection was also used to offer occasional feedback to the staff on progress of the pilot programme. This was eagerly awaited, “Cause obviously we're waiting for that initial data to come back. So yeah, I think we've managed two updates up to now.” (PFG02, PS8) and seen as “really strong” (PFG02, PS5) in terms of effectiveness of the intervention.

Also, coaches found feedback on progress of the pilot programme useful to answer SUs’ questions.

“… participants ask a lot like how can you tell me how it's going? You know what's the being able to go back to him and say look average client loses nine and half kg I think it was or something like that wasn't it?” (PFG02, PS5)

Communication loops were also said to be present for negative/positive feedback and health incidents. When feedback was received from other stakeholders there was a process for it to be disseminated appropriately. If it was positive, it was shared with the team lead and cascaded to team members.

“… we'll share positive feedback, we will contact the delivery coach’s line manager to share positive feedback, to make sure that then gets shared with them. 'Cause it's important that, as line managers, they are aware of their team’s success and feedback that's been provided.” (PFG05, PS21)

If feedback was negative, it was shared with the team lead and shared with team member(s) as appropriate, and followed up, where necessary.

“… in the event we were to get in negative feedback, again we’d follow a similar process so, depending on what the feedback was as well, we would speak to the coach’s line manager or if it was a patient support team, speak to their line manager, or it was to get escalated to myself [senior manager] to investigate, and this could be something like just looking at a training need or it could be absolutely anything.” (PFG05, PS21)

In cases where feedback highlighted a serious issue there was a process to deal with it.

“If it was a severe matter which formed a complaint, we would record this on a separate system. It would still be investigated but where it's recorded, a notification would go out to the other business leads, so it might go to the contract liaison officers, it may then go to [the operations manager]. So, then we've all got a view of what that complaint or incident was and who it, who needs to investigate it. (PFG05, PS21)

It was reported by coaches that there were good feedback loops with SU queries.

“Yeah, massively. I mean myself I know how to speak with all of, I mean not only from the CLO [contract liaison officer] but from the training department, and from the MDT [multidisciplinary team], I mean they're all there to support and just really a click of a button. Never been a wait of two or three days.” (PFG04, PS20)

#### Facilitators to communication

Open channels are the basis for communication. Analysis demonstrated how these require fully operational, complete feedback loops, with efficient and effective communication links that function well. These open channels then equip provider staff to perform to a high standard.

“… we'll just ask a question on there (WhatsApp), or just email another coach and say, do you know anything about this? Have you got any resources?” (PFG2, PS8)

Providers understood the link between acting on feedback and SU benefit.

“So there is opportunity to feed back to one another constantly as well and work together [in regular full team meetings]. So I think all of that, being established from the very beginning, was quite useful, with it being a pilot programme that we'd never delivered on, to actually just figure out things together, and just continuously improve the service, so that, to the patients, they're having a smooth journey when we're onboarding them. So that's how it all came together.” (PFG05, PS24)

Providers also reflected on the importance of these processes in communicating externally, with referrers and funders.

“I think it's about general practice gaining familiarity, really understanding the programme.” (PFG09, PS39)

“It was challenging, and in initially supporting GPs and practitioners to refer in, I think, and that is still, you know there's still apprehension with some of them, but very definitely in the early days supporting that yeah, probably the biggest challenge.” (PFG09, PS39)

Patterns of meetings with referrers varied. Managers from one provider circulated regular, frequent updates and met with other stakeholders at the sites in a structured way, with frequency partly determined by demand.

“… it's quite structured. So, we do weekly reports, monthly reports. Most sites do biweekly site meetings. We and then, you know, so if there's a lot of engagement going on, we can, we will do them weekly. If, if you know if there's not, then they could move it to bi-, you know monthly.” (PFG03, PS13)

#### Barriers to training sessions

There were examples of increased workload as new training sessions had to be developed and standardised. Also, there was additional material to create as the programme became more established.

“… you’ll have a diabetes practitioner coach manual. But usually things like that are all, are all saved online so they can be edited and you’ve got correct live versions as and when.” (PFG05, PS21)

It was clear from the data that the criteria for GP involvement differed across pilot areas (see Additional File 5, Mobilisation) and providers tried to adapt the training material accordingly, however, this created an additional burden e.g. on resources. Providers also referred to how GP services had to learn how to accurately identify and refer suitable patients.

“So we go through that in a lot of detail explaining the referral form process with them and the pathway.” (PFG10, PS49)

Where the planned mode of programme delivery was not online, more training was required to deal with delivery during the pandemic, which had cost and resource implications for providers.

“…a couple of people who were showing us how to work the equipment out on different things, …, and we were going through it and kind of coming up with suggestions around, especially around the book, because on the training, we were literally given like a big A4 booklet part, that we were trying to come up with things on how we could show it to the participant, with us not being there in face …” (PFG01, PS03)

### Within the Collective Action theme

#### Barriers faced by service users

Cultural barriers include Ramadan and Christmas especially during the TDR phase.

“So last year when Ramadan came around, we were actually able to offer patients either, the option to delay their TDR start date, or some of our patients, that were practicing Ramadan, actually were happy to have all four meals within that sunset period. So again, it comes down to kind of personal preference I guess as to how they wanted, but again, they had the option either delay it or have all four meals within that period of time.” (PFG12, PS61)

Veganism was reported as a barrier to the programme because the TDR products are not suitable.

“Vegan TDRs unfortunately are not at our disposal at the moment, so that's one of our biggest challenges. The TDR phase. Going on from there we can, we can do anything. It's the TDR phase that's the limiting factor.” (PFG11, PS57)

Taste preferences were a barrier for some SUs, with providers acknowledging SUs would be keen to test out flavours before ordering.

“There is limited opportunity to swap products, which depends on if providers happen to have a bit of stock leftover in the office. If they’ve got a lot of them, like I said, we do have a little bit of a stock in the Liverpool office, so if there's a lot of them that they don't like, then if we've got something in that we can swap it for it, if they're happy to post it back, we’ll post some out for them. If it's only like a couple of products that people have got, it tends to just, they’ll tend to just use them up at one time.” (PFG01, PS3)

Use of technology can be a barrier for older SUs.

“I have slightly an older clientele on this. Their use of technology is not always the best. So, sometimes, it's making sure you email things across, so they can literally just open it up, rather than them having to find it and going through links, so that's probably the biggest thing.” (PFG01, PS3)

There is an attitude from the coaches that TDR should be acceptable to SUs, albeit difficult, but not something they would willingly accept, which seems incongruous with trust building.

PS61: “I imagine it's difficult at any time. We have all been, I know [PS60] and I have at least anyway, we've actually tried the TDR.

I2: Oh.

PS62: Yeah, they are an interesting variety.

I2: Yeah, I mean just following on from that though. I mean did you, did you try it for the 12 weeks?

PS61: No, absolutely not.” (PFG12)

#### Facilitating referrals

More contact between providers and GP practices was observed to lead to increased referrals.

“Yeah, I was just gonna say kind of like from a referrals’ perspective. Obviously the, this is a pilot programme but we, we have seen you know a jump in referrals and we are seeing more and more patients being referred to us from practices and kind of echoing what [PS25] said there we, we do see that the practices that tend to have more contact with myself or the CLOs directly, they will be kind of sending more patients through to us.” (PFG06, PS16)

### Within the Reflexive Monitoring theme

#### Implementation process

The implementation was reported to involve some challenging and complex processes that highlighted some difficulties in practice.

“… it was challenging . . . you would have to think about though not only the referrals, but the referrals coming through in the right places at the right time so that you don't have to have long waiting times” (PFG03, PS15)

“we've got other key considerations that we need to make around, particularly getting the right venues in the right places that's accessible and these, this whole mini cluster approach”. (PFG09, PS40)

“Making sure that we are, you know, recruiting in a timely manner, having the right amount of people there to deliver the programme”. (PFG10, PS41)

#### Motivation of SUs

Motivation of SUs was seen as crucial to a successful outcome; however individual motivations were reported as changeable. Some SUs appeared to manage well during TDR, but lose motivation on reintroduction of food, and vice versa.

“I think for me it's very, very subjective because some people are really engaged the first 12 weeks and they do brilliantly, but then as they then start to introduce food and they get back onto food then their engagement does just drop. However, you have some people are the complete opposite. They find the first 12 weeks really difficult, and they struggle to really get into the programme. But then something just clicks for them and then they really then just stride through it.” (PFG03, PS11)

Providers reported that SUs needed to be in the right frame of mind and willing to make changes in their lifestyle, otherwise staff thought they tended not to engage properly and were more likely to drop out.

“everybody’s got to be at the right stage of their, of the change, change cycle. And it's it's capturing at that point.” (PFG12, PS67)

“I feel like sometimes you can tell very early on when somebody is going to end up dropping out. You feel like they're not fully engaged.” (PFG04, PS17)

Motivation was reported as easier for those who felt and saw the results quickly.

“if we look at the success of it and what's been working well, we've had so many patients excited at the start when we talked to them originally, and then when they're actually on it and they're achieving what they actually wanted to achieve, because they're putting in the hard work and getting support from us, I think ultimately they’re then very happy with their experiences and want to tell other people to join as well.” (PFG05, PS24)

However, motivation was seen as lower for those living alone or without family support.

“… with my groups you have noticed the difference for those that maybe live alone to those that have family support.” “… they haven't got that support system around them in their own personal environment.” (PFG08, PS37)

If results were less than expected, motivation was observed to lower.

**“…** the 12 weeks was really hard, and they would have expected a bit more weight loss, even though they’ve still lost a couple of stone and, and in the kind of 12 weeks”. (PFG01, PS03)

Anecdotally, drop-out rate tended to be higher in the groups mode compared to the 1:1.

“We had a few participants like that who the group setting didn't really work [for] and they would have benefited more from a one-to-one support.” Some participants were unable to follow through with certain activities or the take home tasks. “I don't know whether that's because of difference in education level or anything like that. But they just needed a bit more support than others.” (PFG08, PS36)

#### Internal feedback

Provider staff identified that regular feedback and updates needed to be prioritised, so that they were structured in regularly and available at least monthly.

“Maybe just a bit more kind of regular” (PFG01, PS03)

Although there was a recognition of the difficulties of bringing everyone together for feedback and update sessions, staff felt this was important.

“Trying to get everybody, where they are free, at the same time, is really difficult, to squeeze everyone in.” (PFG01, PS03)

Offering individual catch-up sessions for frontline staff, on a one-to-one basis if they can’t attend group sessions, was seen as important in ensuring more staff receive feedback and updates.

“More regular check-ins, possibly one to ones” (PFG01, PS01)

Recognising the importance of communicating progress across the programme staff and knowing how others are doing were also valued.

“… useful to have a bit of inclination of how others are getting on” (PFG01, PS01)

## REFERENCE

May, C.*, et al.* (2007) 'Understanding the implementation of complex interventions in health care: the Normalization Process Model', *BMC Health Services Research,* 7(148).

## Information on training sessions

| **Training** | **Components** |
| --- | --- |
| Standard | Structure predetermined by NHSE |
|  | Providers created their own materials |
|  | Standard on-boarding includes core elements of the programme |
|  | Content varies between providers but has similar elements e.g. behaviour change |
|  | Training provided in different skills e.g. motivational interviewing |
|  | Designed to be tailored by frontline staff to individual SUs |
| Additional  Methods | Responsive to need, where gaps in knowledge were exposed e.g. cultural awareness |
|  | Basic training sessions |
|  | Interactive role play |
|  | Skills training |
|  | Observing others |
|  | Listening in to others |
|  | Shadowing others |
|  | Monitoring staff competency and progress |
|  | Shadowing |
|  | Learning on the job |
